# Mn‐Substituted Tunnel‐Type Polyantimonic Acid Confined in a Multidimensional Integrated Architecture Enabling Superfast‐Charging Lithium‐Ion Battery Anodes

**DOI:** 10.1002/advs.202002866

**Published:** 2020-11-25

**Authors:** Boya Wang, Yunhong Wei, Haoyu Fang, Xiaoling Qiu, Qiaobao Zhang, Hao Wu, Qian Wang, Yun Zhang, Xiaobo Ji

**Affiliations:** ^1^ Department of Advanced Energy Materials College of Materials Science and Engineering Sichuan University Chengdu Sichuan 610064 P. R. China; ^2^ Department of Materials Science and Engineering College of Materials Xiamen University Xiamen Fujian 361005 P. R. China; ^3^ College of Chemistry and Chemical Engineering Central South University Changsha Hunan 410083 P. R. China

**Keywords:** element substitution, fast charging anode, lithium‐ion batteries, multidimensional architecture, polyantimonic acid

## Abstract

Given the inherent features of open tunnel‐like pyrochlore crystal frameworks and pentavalent antimony species, polyantimonic acid (PAA) is an appealing conversion/alloying‐type anode material with fast solid‐phase ionic diffusion and multielectron reactions for lithium‐ion batteries. Yet, enhancing the electronic conductivity and structural stability are two key issues in exploiting high‐rate and long‐life PAA‐based electrodes. Herein, these challenges are addressed by engineering a novel multidimensional integrated architecture, which consists of 0D Mn‐substituted PAA nanocrystals embedded in 1D tubular graphene scrolls that are co‐assembled with 2D N‐doped graphene sheets. The integrated advantages of each subunit synergistically establish a robust and conductive 3D electrode framework with omnidirectional electron/ion transport network. Computational simulations combined with experiments reveal that the partial‐substitution of H_3_O^+^ by Mn^2+^ into the tunnel sites of PAA can regulate its electronic structure to narrow the bandgap with increased intrinsic electronic conductivity and reduce the Li^+^ diffusion barrier. All above merits enable improved reaction kinetics, adaptive volume expansion, and relieved dissolution of active Mn^2+^/Sb^5+^ species in the electrode materials, thus exhibiting ultrahigh rate capacity (238 mAh g^−1^ at 30.0 A g^−1^), superfast‐charging capability (fully charged with 56% initial capacity for ≈17 s at 80.0 A g^−1^) and durable cycling performance (over 1000 cycles).

## Graphical abstract

Confining 0D Mn^2+^‐substituted tunnel‐type polyantimonic acid nanocrystals in a 1D tubular graphene scrolls assembled with 2D graphene sheets builds up a novel 0D⊂1D⊂2D multidimensional integrated architecture with enhanced electronic/ionic transport kinetics and adaptive volume expansion, enabling superfast‐charging and long‐life lithium‐ion battery anodes.

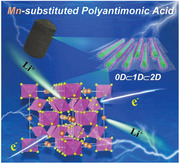

## 1 Introduction

High‐rate lithium‐ion batteries (LIBs) with fast charging feature and high energy density are urgently needed for mobile electronics and electric vehicles. Nevertheless, the energy/power densities of LIBs constructed by the conventional intercalation‐type graphite and Li_4_Ti_5_O_12_ anodes have reached to their theoretical limit.^[^ ^1^, ^2^, ^3^ ^]^ In recent years, some emerging anode materials such as silicon, phosphorus, and lithium metal have been extensively researched aiming to increase the energy density of LIBs, while little focus has been given on boosting the fast‐charging property of batteries.^[^ ^4^, ^5^, ^6^, ^7^, ^8^, ^9^ ^]^

Polyantimonic acid (PAA, H_2_Sb_2_O_6_ ·*n*H_2_O) is an appealing conversion/alloying‐based anode material for LIBs due to the advantages of low cost, suitable reaction potential, high theoretical specific capacity endowed by pentavalent Sb^5+^ species, and fast solid‐phase ionic diffusion associated with its three‐dimensional (3D) open tunnel‐like pyrochlore crystal framework.^[^ ^10^, ^11^ ^]^ Very recently, we have demonstrated for the first time that PAA composited with conductive graphene, served as a conversion/alloying‐based anode, is able to exert high electrochemical activity for reversible Li^+^‐ and K^+^‐ion storage.^[^ ^10^ ^]^ Nevertheless, the application of PAA anodes for fast‐charging and long‐cycling LIBs is still restricted by two key issues. On the one hand, the inherent low bulk conductivity of PAA cannot be substantially enhanced via the simple compositing with 2D planar graphene, leading to a limited reaction kinetics associated with the constrained charge transfer in PAA. On the other hand, despite achieving a good electrochemical reversibility, the open architecture of the composites is not able to fully confine the volume changes and self‐aggregation of PAA particles during the conversion and alloying process, which caused the structural degradation, and eventually resulted in capacity decay after long cycles.

As an efficient strategy in modulating crystal structure and electronic structure of various inorganic materials,^[^ ^12^, ^13^, ^14^ ^]^ element substitution/doping could be a viable way to tackle the poor inherent electronic conductivity of the PAA. Fortunately, PAA, owning a pyrochlore‐phase crystal framework, allows a good flexibility in compositional tuning, which is in favor of the element doping/substitution.^[^ ^15^, ^16^, ^17^ ^]^ From the structural viewpoint, pyrochlore PAA is built up of corner‐sharing SbO_6_ octahedra that establish a 3D (Sb_2_O_6_^2−^) framework, in which interconnected tunnel‐like structure with a large hydronium (H_3_O^+^)‐containing cavity exist. Such open tunnels are in favor of proton mobility (10^−3^–10^−4^ S cm^−1^) under the “Grotthuss mechanism” and especially the ion exchange process with various size metal cations, such as Li^+^ (0.076 nm), Na^+^ (0.102 nm), Mn^2+^ (0.083 nm), Co^2+^ (0.065 nm), and Ni^2+^ (0.069 nm).^[^ ^18^, ^19^, ^20^, ^21^, ^22^ ^]^ Actually, PAA is often used as a superior inorganic ion‐exchanger because of high‐efficiency cation exchange capability with favorable ion selectivity.^[^ ^23^, ^24^, ^25^ ^]^ Thanks to this specific property, it enables the element doping/substitution into the tunnel sites of PAA crystal framework, while making it feasible to yield a modulation on its physicochemical properties.^[^ ^11^, ^22^, ^26^ ^]^ For example, Ozawa prepared bismuth‐substituted PAA (H_1‐3_ *_x_*Bi*_x_*SbO_3_ ·*n*H_2_O) through a solution processing, and they founded that the Bi‐substitution into the tunnel sites can greatly enhance the proton conductivity of PAA with almost two orders of magnitude.^[^ ^27^ ^]^ Other than Bi, yttrium‐substituted PAA (H_1‐3_ *_x_*Y*_x_*SbO_3_ ·*n*H_2_O) is also proved to possess a higher proton conductivity (3.8 × 10^−3^ S cm^−1^) than pristine PAA (8.3 × 10^−5^ S cm^−1^).^[^ ^28^ ^]^ In addition, the spin glass behavior or magnetic property of pyrochlore PAA‐based materials is also adjustable by substituting with different elements (e.g., Mn, Co, or Ni).^[^ ^26^, ^29^, ^30^ ^]^ Thereby, it is very likely to boost the inherent electronic conductivity of PAA by regulating its crystal or electronic structure through the substitution of metal cations over the H_3_O^+^ in the tunnel cavity of PAA. More notably, the ionic migration behavior in the open tunnel framework of PAA could be also changed along with the modulated structural properties. To date, however, there is no report regarding the structure regulation on PAA‐based electrode materials via element substitution/doping, and the resulting effects to electrochemical energy storage kinetics still remain unexplored.

In addition to elevating the inherent electronic conductivity, construction of robust and high‐stable electrode structures is also urgently required to ensure a durable cycling stability of PAA anode. Although building an crosslinked electrode framework using planar graphene can meet the abovementioned requirement to some extent,^[^ ^10^ ^]^ the incomplete encapsulation by 2D graphene sheets still leads to a significant volume variation of PAA particles during the ion (de)intercalation process. As a new blood of the graphitic family, graphene scroll (GS), which is formed by scrolling 2D graphene sheets, has triggered great research attention owing to its unique 1D topology structure with open interior spaces.^[^ ^31^, ^32^ ^]^ The capability of wrapping foreign molecules into the interior cavities of tubular GS together with a topological space confinement has been also demonstrated by theoretical calculations and more importantly, practical applications in various electrochemical energy storage.^[^ ^32^, ^33^, ^34^ ^]^ In our previous work, for instance, porous MnO nanowires sheathed in 1D GS with internal voids was prepared through a facile synthesis protocol, which exhibits a significantly restrained volume expansion and long‐term cycling stability in LIBs.^[^ ^35^ ^]^ Inspired by this, a robust and stable PAA‐based electrode structure is also expected to be achieved via encapsulation and confinement of the PAA into the tubular GS, so as to circumvent the issue of structural degradation caused by the large volume expansion.

To simultaneously address the challenges in enhancing the electronic conductivity and structural stability of PAA, herein, we report the structural engineering of a novel multidimensional integrated architecture, which consists of 0D Mn‐substituted PAA nanocrystals (Mn‐PAA) embedded in 1D tubular graphene scrolls that are co‐assembled with 2D nitrogen‐doped graphene sheets. Building such a multidimensional integrated architecture (denoted as Mn‐PAA⊂GS⊂G) is based on a hydrothermally assisted smart strategy called as “kill two birds with one stone,” which relies on employing MnO_2_ nanowires both as a manganese source for Mn‐substitution in PAA crystal and a template for directionally inducing the formation of GS. Experimental results together with theoretical calculations disclose that the partial substitution of H_3_O^+^ by Mn^2+^ into the tunnel cavities of PAA is able to regulate its electronic structure, which results in narrowed bandgap with exponentially increased intrinsic electronic conductivity and reduced migration energy barrier of Li^+^ ions in the open tunnels of PAA. More importantly, the well‐defined multidimensional assembled architecture (i.e., 0D⊂1D⊂2D) combined with advantages of each 0D, 1D, and 2D subunit establishes a unique robust 3D electrode framework with an omnidirectional conductive network for electron/ion transfer. Furthermore, the confinement by GS with rich internal space can accommodate the repeated volume changes of the enclosed Mn‐substituted PAA nanocrystals, and relieve the dissolution/loss of active Mn^2+^/Sb^5+^ species from the electrode materials. Benefiting from the synergistic effects on improving the electronic conductivity, ion mobility, and structural stability of tunnel‐type PAA, the as‐built Mn‐PAA⊂GS⊂G electrode exhibits a high reversible capacity (719 mAh g^−1^ at 0.1 A g^−1^), extraordinary rate performance (238 mAh g^−1^ at 30.0 A g^−1^), superfast‐charging capability (56% of initial capacity charged in ≈17 s at 80.0 A g^−1^), and durable long cycling life (1077 mAh g^−1^ after 1000 cycles at 1.0 A g^−1^).

## 2 Results and Discussion

**Figure 1:**
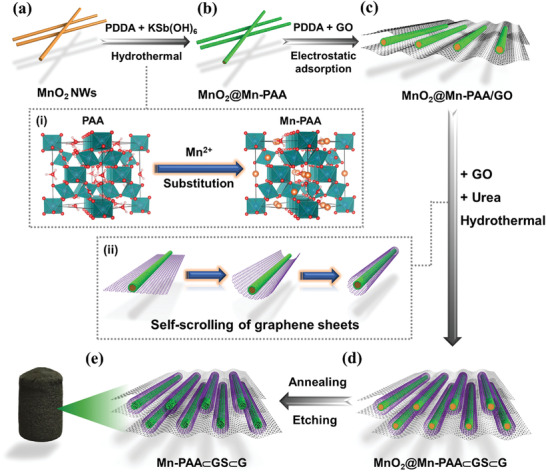
Schematic illustration of the fabrication process of the 3D Mn‐PAA⊂GS⊂G architecture.

**Figure 2:**
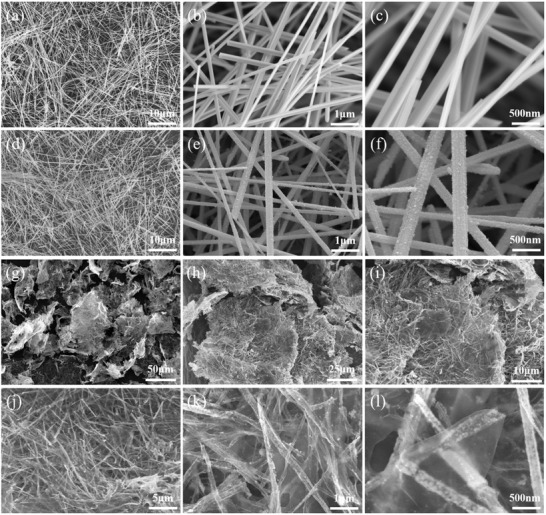
FESEM images of the a–c) MnO_2_, d–f) MnO_2_@Mn‐PAA, and g–l) Mn‐PAA⊂GS⊂G.

The synthetic process is illustrated in **Figure** **1**, and the brief photograph exhibition is presented in Figure S1 (Supporting Information). First, ultralong *α*‐MnO_2_ nanowires (Figure S2, Supporting Information) with a large aspect ratio over 1000 were synthesized by a simple hydrothermal method (Figures 1a and **2**a–c). Then, the MnO_2_ nanowires were surface‐modified by a cationic polyelectrolyte (poly(diallyldimethylammonium chloride), PDDA) to become positive charged, so as to react with the Sb precursor, potassium hexahydroantimonate (V) (KSb(OH)_6_, Figure S3, Supporting Information). With the aid of hydrothermal method, it can simultaneously achieve the uniform growth of PAA nanocrystals on the surface of MnO_2_ nanowires (Figure 1b) and the Mn‐substitution in the tunnel sites of the resulted PAA crystal structure (Figure 1i; Figure S4, Supporting Information). The positively charged surfaces of the PDDA‐modified MnO_2_ nanowires are in favor of the adsorption of Sb(OH)_6_^−^ anions through electrostatic interaction (Figure S5, Supporting Information), thus facilitating the efficient nucleation and homogeneous crystal growth of PAA (Figure 2d–f). Without the employment of PDDA, for comparison, the deposition of PAA is not uniform with irregular particle sizes (Figure S6, Supporting Information).

After further surface‐modified with PDDA, the positively charged core‐shell MnO_2_@H_2‐_ *_x_*Mn*_x_*Sb_2_O_6_·*n*H_2_O composite nanowires (MnO_2_@Mn‐PAA) were then mixed with negatively charged graphene oxide (GO) to afford a close and uniform connection under the drive of electrostatic attraction (MnO_2_@Mn‐PAA/GO) (Figure S7, Supporting Information). The obtained MnO_2_@Mn‐PAA/GO was then homogeneously dispersed in GO/urea aqueous solution. After experienced a hydrothermal reaction, the GO were gradually reduced and doped with N owing to the existence of urea, whilst triggered the rolling up of the reduced GO sheets along the composite nanowire surface (Figure 1ii) to form a core‐sheath nanocable (MnO_2_@Mn‐PAA⊂GS). Owing to the *π*‐*π* bonding and hydrophobic effects between the graphene sheets, the MnO_2_@Mn‐PAA@GS nanocables can be crosslinked with additional graphene sheets, and then self‐assembled into a 3D hydrogel (MnO_2_@Mn‐PAA⊂GS/G, Figure 1d; Figure S8, Supporting Information). After freeze‐drying, annealing, and removing the MnO_2_ core by selective etching, the final 3D macroscopic foam‐like Mn‐PAA⊂GS⊂G is obtained (Figure 1e). The key of the whole assembly process is to prepare the core‐sheath coaxial MnO_2_@Mn‐PAA@GS nanocables, which can be smartly accomplished by adopting 1D MnO_2_ nanowires to serve both as a manganese source and a template in a two‐step hydrothermally assisted Mn‐substitution and graphene self‐scrolling processes. In the first hydrothermal process (Figure 1a,b), under a high‐temperature and high‐pressure atmosphere, the MnO_2_ nanowires that have a very high surface activity can react with the solvent molecules, resulting in that a small quantity of reduced Mn^2+^ could be released from the nanowire surface (Figure S9, Supporting Information) and involve in the partial substitution into the tunnel sites of PAA crystal structure (Figure 1i). In the second hydrothermal treatment (Figure 1c,d), the attractive interaction between graphene sheets and MnO_2_@Mn‐PAA composite nanowires can drive some adhered graphene sheets to bend along the nanowire in the radial direction, and then the bent graphene sheets further scroll surrounding the nanowire, leading to the consequent formation of the nanowire‐templated GS architecture (Figure 1ii).

The morphologies of the Mn‐PAA⊂GS⊂G and its control samples (see details in Experimental Section, Supporting Information) were firstly measured by field‐emission scanning electron microscopy (FESEM). The Mn‐PAA⊂GS⊂G is composed of 2D large‐area nest‐like graphene sheets, as demonstrated in Figure 2g–i. Through higher magnification FESEM images (Figure 2j–l), interconnected 1D tubule‐shaped GS is deeply embedded in and throughout the 2D graphene sheet networks. Meanwhile, there are many tiny 0D PAA nanocrystals scattered but well encapsulated in the GS with a peapod‐like structure, unveiling the multidimensional assembled, and hierarchical architecture of the Mn‐PAA⊂GS⊂G. Notably, there are rich internal voids formed in the Mn‐PAA⊂GS after the selective removal of MnO_2_ nanowire cores (Figure 2k,l). In contrast, Mn‐substituted PAA without graphene (denoted as Mn‐PAA) is mostly made of numerous irregular and aggregated particles with varying sizes (Figure S10a–c, Supporting Information), but they are still quite different from the larger‐sized bare PAA polyhedron (Figure S11, Supporting Information). For the Mn‐PAA/G that was prepared by mechanically mixing Mn‐PAA with graphene, it can be observed that aggregated Mn‐PAA particles are randomly attached on the surface of the graphene sheets (Figure S10d–f, Supporting Information). Moreover, N_2_ adsorption/desorption measurements (Figure S12, Supporting Information) also uncover that the Mn‐PAA⊂GS⊂G, having a more developed mesoporous structure, shows a larger Brunauer–Emmett–Teller (BET) specific surface area of 90.1 m^2^ g^−1^ and a higher total pore volume of 0.341 cm^3^ g^−1^ (Table S1, Supporting Information), which is much higher than those of the Mn‐PAA (10.6 m^2^ g^−1^; 0.076 cm^3^ g^−1^) and Mn‐PAA/G (22.2 m^2^ g^−1^; 0.133 cm^3^ g^−1^).

**Figure 3:**
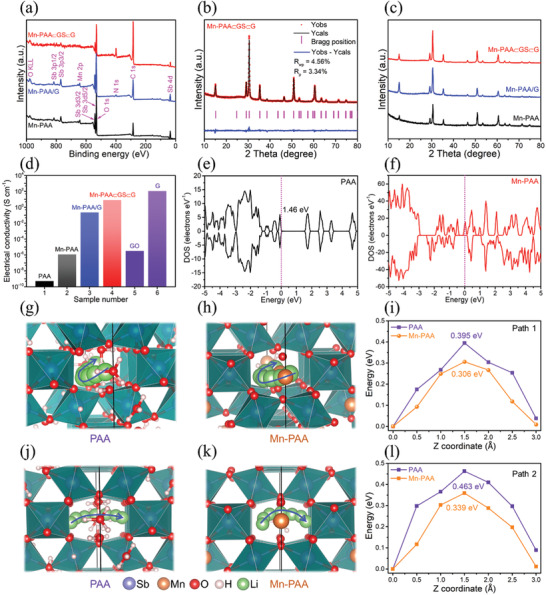
a) XPS spectra of the Mn‐PAA, Mn‐PAA/G, and Mn‐PAA⊂GS⊂G; b) Rietveld refinement patterns of the Mn‐PAA⊂GS⊂G; c) XRD patterns of the Mn‐PAA, Mn‐PAA/G, and Mn‐PAA⊂GS⊂G; d) Electrical conductivity of samples; Calculated DOS for the e) PAA and f) Mn‐PAA; Migration pathway and diffusion energy barrier of Li^+^ ion through g–i) Path 1 and j–l) Path 2 in the tunnel of bare PAA (g, j) and Mn‐PAA (h, k).

X‐ray photoelectron spectroscopy (XPS) was adopted to characterize the composition and chemical state of all the samples. The survey scan XPS profiles of the Mn‐PAA (**Figure** **3**a) certify the existence of Mn, Sb, O, and C elements with no evidence of other impurities. Compared with the Mn‐PAA, there are obvious signals of N element in the Mn‐PAA/G and Mn‐PAA⊂GS⊂G, which are derived from the nitrogen doping in the graphene matrix. As for the Mn‐PAA⊂GS⊂G, it has significantly increased signals from C and N along with decreased ones from Mn and Sb, due to the full encapuslation of Mn‐PAA by GS⊂G. The Sb 3d spectrum of the Mn‐PAA⊂GS⊂G (Figure S13a, Supporting Information) exhibits two characteristic peaks at 530.9 and 540.2 eV, corresponding to the Sb 3d_5/2_ and Sb 3d_3/2_ of pentavalent Sb^5+^ species respectively.^[^ ^10^, ^21^, ^36^ ^]^ Besides, there are two O 1s peaks resolved at 531.3 and 533.4 eV for the Mn‐PAA⊂GS⊂G, which are attributed to the oxygen components seperately originated from the Mn‐PAA and the GS⊂G.^[^ ^36^ ^]^ More notably, the Mn 2p spectrum of the Mn‐PAA⊂GS⊂G is obtained (Figure S13b, Supporting Information), and it can be resolved into two main peaks situated at 641.7 (Mn 2p_3/2_) and 653.8 eV (Mn 2p_1/2_), corresponding to the Mn^2+^, which confirms the existence of divalent manganese in the Mn‐PAA⊂GS⊂G.^[^ ^37^, ^38^ ^]^ The two N 1s peaks of the Mn‐PAA⊂GS⊂G can be ascribed to pyridinic‐N (398.8 eV) and pyrrolic‐N (400.2 eV) (Figure S13c, Supporting Information), and the N‐doping is believed to be beneficial for enhancing the electronic conductivity of the graphene and improving its adsorption ability to active species.^[^ ^39^, ^40^, ^41^, ^42^ ^]^ Elemental analysis (EA) results reveal that the total contents of C and N in the Mn‐PAA/G and Mn‐PAA⊂GS⊂G are 45.9% and 45.4%, respectively (Table S2, Supporting Information).

Raman spectrum was recorded to further study of the nature of carbon in the Mn‐PAA⊂GS⊂G. As presented in Figure S14 (Supporting Information), two peaks situated at ≈1347 and ≈1600 cm^−1^ are originated from the D band (defective structure) and G band (graphitic structure), respectively.^[^ ^43^, ^44^ ^]^ Obviously, the relatively higher *I*_D_/*I*_G_ intensity ratio for the Mn‐PAA⊂GS⊂G (1.09) compared with the GO (*I*_D_/*I*_G_ = 0.98) indicates the more defective sites in the reduced GO for the Mn‐PAA⊂GS⊂G upon the N doping.^[^ ^35^ ^]^ The Raman spectra of bare PAA and Mn‐PAA were also tested and exhibited in Figure S15 (Supporting Information). The bare PAA shows four Raman vibration peaks in the range of 200–1000 cm^−1^, which can be assigned to Sb–O vibrations (443, 509, and 615 cm^−1^) and O–O stretching (854 cm^−1^).^[^ ^10^, ^23^, ^45^ ^]^ The Mn‐PAA presents similar Raman spectrum with the bare PAA, suggesting that they share similar structure. However, it should be noted that there is an obvious blue shift for the Sb‐O stretching (PAA: 509 cm^−1^; Mn‐PAA: 522 cm^−1^). In addition, the introduction of Mn^2+^ also caused the changes of peak intensities, especially for the Sb–O vibrations at 289 and 443 cm^−1^. These changes on the Raman spectra of the PAA caused by Mn^2+^ doping are similar with other metal ions‐doped material systems.^[^ ^46^, ^47^, ^48^ ^]^

To identify the crystal structure and phase purity, X‐ray diffraction (XRD) technique along with the Rietveld refinements were performed for the Mn‐PAA⊂GS⊂G (Figure 3b) and bare PAA (Figure S16, Supporting Information). The XRD refinement were successfully implemented using a crystallographic model of cubic pyrochlore polyantimonic acid reference (COD ID 1529908), and a reasonable fit with good reliability factors (*R*_wp_ < 7%; *R*_p_ < 5%) can be obtained. While the lattice parameters obtained by Rietveld refinement are *a* = *b* = *c* = 10.333 Å for the bare PAA, and *a* = *b* = *c* = 10.162 Å for the Mn‐PAA⊂GS⊂G, both of the two samples show identical cubic pyrochlore phase with a space group symmetry of Fdm (Table S3, Supporting Information). This implies that the Mn incorporation does not lead to a transformation of the pyrochlore structure of PAA. However, the relatively smaller lattice parameters of the Mn‐PAA⊂GS⊂G than that of the bare PAA suggests that the Mn‐substitution leads to a slight contraction of the unit cell volume, which is also reflected by the slight shift of diffraction angle (2*θ*) to a higher position after the Mn introduction. The decrease of the unit cell volume may be ascribed to the incorporated Mn^2+^ ions that link with the negatively charged Sb_2_O_6_^2−^ units, suggesting the plausible doping of Mn^2+^ into tunnel sites of the PAA. Generally speaking, the lattice cell of pyrochlore‐type PAA has four atomic occupancy positions, namely, 16d, 16c, 48f, and 8b sites. Specifically, the centers of SbO_6_ octahedra are occupied by Sb^5+^ ions (16c site), the centers of cubes contain H_3_O^+^ cations (16d site), the apices of octahedra correspond to O^2–^ (48f site), and their junction points are occupied by H_2_O (8b site). By comparing the ionic radius size, Mn^2+^ (*r* = 83 pm) is much larger than Sb^5+^ (*r* = 60 pm) when they share the same coordination number of 6. Therefore, given the huge difference in charge and ionic radius between Mn^2+^ and Sb^5+^ as well as the change of the lattice parameters, one can speculate that the Mn^2+^ ions in the Mn‐PAA are not substituted for the Sb^5+^ ions to occupy the 16c site, but placed on the interstitial sites in the tunnel cavities of PAA, i.e., the 16d site. The Rietveld refinement results also confirmed that the Mn^2+^ ions cannot occupy the 16c site (Figure S17, Supporting Information). Indeed, except for the 16d site, the Mn^2+^ ions can be also assumed to occupy the 8b site. Nonetheless, we found that such positioning led to poorer convergence in the Rietveld refinement results (Figure S18, Supporting Information). When the Mn^2+^ ions are fixed at the 16d sites, the amount of proton would be decreased to keep charge balance, and hence, the compositional formula could be speculated to be H_2‐2_ *_x_*Mn_2_ *_x_*Sb_2_O_6_O*_x_*·*m*H_2_O (*m* < *n*) for the Mn‐substituted PAA. More specifically, according to the atomic occupancy ratio obtained from the Rietveld refinements (Table S3, Supporting Information), the *x* value in the Mn‐substituted PAA is determined to be 0.81, namely H_0.38_Mn_1.62_Sb_2_O_6_O_0.81_·*m*H_2_O. Furthermore, the inductively coupled plasma optical emission spectroscopy (ICP‐OES) test also verifies that the mole ratio of Mn and Sb in Mn‐substituted PAA is calculated to be around 1.60: 2 (Table S4, Supporting Information), which is in accordance with the conclusion of EDX test (1.57: 2, Figure S19, Supporting Information). After the substitution of Mn^2+^ for O(H_3_O^+^) in the 16d position, the contents of H_2_O would be reduced. The bare PAA and Mn‐PAA were tested by thermogravimetric analysis and the results were shown in Figure S20 (Supporting Information). Compared with bare PAA (15.3%), the weight loss of the Mn‐PAA is less (7.1%), proving the substitution of Mn^2+^. As for the other two control samples, Mn‐PAA and Mn‐PAA/G, both of their XRD patterns are well indexed to the pyrochlore phase, which are almost identical to that of Mn‐PAA⊂GS⊂G (Figure 3c), indicating the high purity of all the Mn‐substituted PAA samples.

Electrochemical reversibility and reaction kinetics of electrodes are highly dependent on electronic conductivity of active electrode materials. Accordingly, the electronic conductivities of all the samples were first compared. As demonstrated in Figure 3d, the bare PAA has a very poor electronic conductivity as low as 5.2 × 10^−10^ S cm^−1^, which is related to the highest oxidation state Sb(V). As compared with bare PAA, the electronic conductivity of the Mn‐PAA significantly increases to 1.2 × 10^−6^ S cm^−1^, almost four orders of magnitude higher than that of the bare PAA. This fact demonstrates that the substitution of Mn^2+^ is able to substantially improve its inherent bulk electron conductivity, which could be resulted from the beneficial tuning on the electronic structure of PAA. Thus, density functional theory (DFT) calculations were further employed to predict the electronic structure variation of PAA after the introduction of Mn^2+^. Figure 3e,f presents the density of states (DOS) of the bare PAA and Mn‐PAA. For the bare PAA, the conduction band and the valence band were clearly separated by Fermi level with an energy gap of ≈1.46 eV. However, the band gap of Mn‐substituted PAA is drastically narrowed because of the increased DOS in the vicinity of Fermi level, suggesting its higher electronic conductivity.^[^ ^49^, ^50^ ^]^

The narrowed band gap can be also reflected by the optical adsorption properties of the PAA and Mn‐PAA measured by ultraviolet–visible diffuse reflectance spectra (UV–vis DRS) technique. From their absorption spectra (Figure S21a, Supporting Information), the Mn‐PAA shows a higher light harvesting ability in the spectral region of 200–800 nm compared with the bare PAA that only adsorb UV light. A red shift phenomenon of the absorption edge form UV region to visible region can be observed after the substitution of Mn^2+^, which is consistent with the color change of PAA from white to brown (inset in Figure S18a, Supporting Information). According to the relationships between (*αhν*)^1⁄n^ and *hv* based on Kubelka–Munk equation, as shown in Figure S21b (Supporting Information), Mn^2+^ substitution would reduce the band gap energy of PAA from 3.92 to 0.97 eV, thus bestowing the enhanced electronic conductivity of the Mn‐PAA. This is in good agreement with the prediction from DFT calculations. After compositing with the highly conductive reduced GO matrix, a further increase in the electronic conductivity can be achieved for the Mn‐PAA/G (0.23 S cm^−1^) and the Mn‐PAA⊂GS⊂G (8.2 S cm^−1^), respectively.

In addition to the energy band structure, the possible Li^+^ ions diffusion pathways when intercalated into the tunnel structure for the bare PAA and Mn‐substituted PAA were also simulated. There are two possible Li^+^ migration pathways proposed for both the PAA and the Mn‐PAA, which are denoted as Path 1 (Figure 3g,h) and Path 2 (Figure 3j,k), respectively. The migration energy barriers of Li^+^ ions were calculated by the climbing nudged elastic band (CI‐NEB) methods. As shown in Figure 3i, the energy barrier is 0.306 eV when Li^+^ ion diffuses along the Path 1 in the tunnel framework for the Mn‐PAA, which is smaller than that of the bare PAA (0.395 eV). As for the Path 2 (Figure 3l), a similar comparison result can be also obtained between the Mn‐PAA (0.339 eV) and the bare PAA (0.463 eV). Apparently, the Mn‐PAA exhibits lower Li^+^ migration barriers compared with the bare PAA for both pathways, elucidating that the diffusion of Li^+^ ions in the open pyrochlore crystal frameworks is facilitated after the substitution of H_3_O^+^ by Mn^2+^. Besides, it is worthy to mention that all the calculated energy barriers are less than 0.5 eV, implying a boosted ionic conductivity would be achieved in the pyrochlore Mn‐substituted PAA based electrode materials.^[^ ^50^ ^]^

**Figure 4:**
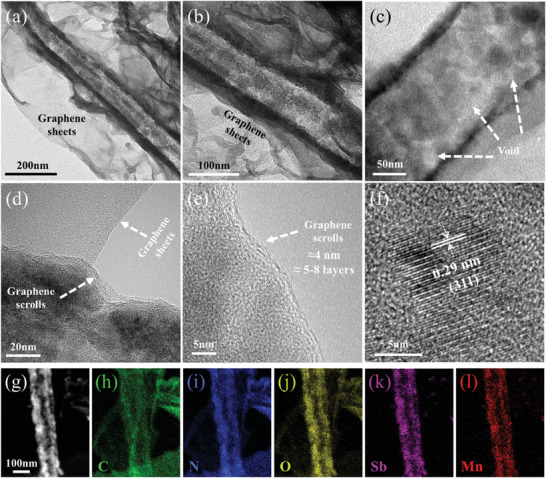
a–e) TEM and f) HRTEM images of the Mn‐PAA⊂GS⊂G; g–l) High‐angle annular dark field scanning TEM image and corresponding EDX element mapping images of the Mn‐PAA⊂GS⊂G.

The microstructure of the Mn‐PAA⊂GS⊂G was researched by transmission electron microscopy (TEM). As demonstrated in **Figure** **4**a,b, there is a typical tubular structure unit that is composed of Mn‐PAA wrapped by 1D GS, which are further embedded in 2D crosslinked graphene sheet network. A magnified TEM image in Figure 4c clearly reveals that, as a result of the removal of MnO_2_ nanowires template, the porous structure of the 1D GS with rich internal voids comprises dispersed nanocrystals with diameter of 15–30 nm. Such an intriguing structure is very helpful for ensuring the electrolyte infiltration and accommodating the volume expansion during electrochemical reaction. As shown in Figure 4d,e, the external GS sheaths can be clearly observed and the thickness is measured to be ≈4 nm, nearly equivalent to 5–8 layers (Figure 4e). Meanwhile, the explicit lattice fringes of one single nanocrystal present the interplanar spacing of 0.29 nm (Figure 4f), well corresponding to the (311) planes of pyrochlore phase PAA. The energy dispersive X‐ray (EDX) line scan result (Figure S22, Supporting Information) proves that the Mn‐PAA nanocrystal core is fully wrapped by the GS shell. More notably, there is a similar curve shape for the Sb and Mn element except the relatively lower intensity of Mn, suggesting that the Mn element was successfully doped into the PAA. The EDX elemental mapping (Figure 3g–l) further indicates the distribution states of the Mn, Sb, C, O, and N elements in the Mn‐PAA⊂GS⊂G, in which Mn and Sb are mainly distributed along the axial direction of the 1D GS. To rule out the possibility that the coating layer maybe derived from PDDA, a MnO_2_@Mn‐PAA sample suffered PDDA treatment was annealed at 400 °C for 2 h in argon atmosphere and characterized by TEM technique. As shown in Figure S23 (Supporting Information), the MnO_2_@Mn‐PAA presents 1D structure (Figure S23a–c) but there is no carbon layer can be detected on its surface (Figure S23d,e). The EDX line scanning results (Figure S23f) also shows that the carbon signal is very weak. EA reveals that the carbon content for the MnO_2_@Mn‐PAA sample is only 0.25 wt%. These results verify that the coating layer is constructed by the multilayered graphene scrolls.

**Figure 5:**
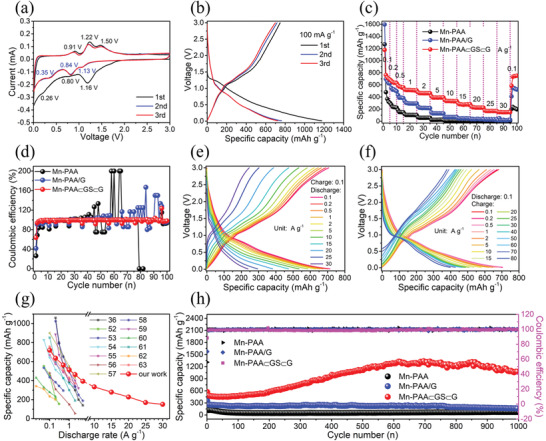
a) CV curves for the Mn‐PAA⊂GS⊂G at a scan rate of 0.1 mV s^−1^; b) Initial three discharge/charge profiles of the Mn‐PAA⊂GS⊂G; c) Rate capability and d) Coulombic efficiency of the Mn‐PAA, Mn‐PAA/G, and Mn‐PAA⊂GS⊂G; e) Discharge/charge profiles of the Mn‐PAA⊂GS⊂G at a fixed charge rate of 0.1 A g^−1^ with discharge rates changing from 0.1 to 30 A g^−1^; f) Discharge/charge profiles of the Mn‐PAA⊂GS⊂G at a fixed discharge rate of 0.1 A g^−1^ with charge rates changing from 0.1 to 80 A g^−1^; g) Comparison of the rate capability of Mn‐PAA⊂GS⊂G with some previously reported Sb‐O based electrodes; h) Long cycling performance of the Mn‐PAA, Mn‐PAA/G, and Mn‐PAA⊂GS⊂G at 1 A g^−1^.

The lithium storage performance of the Mn‐PAA⊂GS⊂G electrode was evaluated using standard coin‐type cell configuration. **Figure** **5**a exhibits the typical cyclic voltammetry (CV) curves of the Mn‐PAA⊂GS⊂G in the initial three cycles. In the first cathodic scan, it shows two peaks at around ≈1.16 and 0.80 V, and they gradually stabilize at ≈1.13 and 0.84 V in the following scans, which is related to the lithiation of PAA to form metallic Sb and Li_2_O and the alloying reaction between Sb and Li forming the Li*_x_*Sb alloy. The difference between the first and subsequent cycles could be ascribed to a surface/structure activation process of electrode materials accompanied with the formation of solid electrolyte interfaces (SEI) layer.^[^ ^10^, ^36^ ^]^ Note that there is another weak cathodic peak at ≈0.25 V, and it shifts to 0.35 V in the following cycles. Given that the Mn‐PAA contains Mn^2+^ that is also an electrochemically active species, it is reasonable to speculate that this cathodic peak could be ascribed to the reduction of Mn^2+^ to metallic Mn^0^. This can be corroborated by comparing the CV curves of the bare PAA, it shows no any cathodic peaks below 0.4 V during the cathodic scan (Figure S24, Supporting Information). For the first anodic scan, the peak at 0.91 V is attributed to the dealloying reaction of the Li*_x_*Sb to become Sb, while the other two oxidation peaks at 1.22 and 1.50 V correspond to the further oxidation conversion of metallic Mn and Sb.^[^ ^36^, ^51^, ^52^ ^]^ For comparison, the CV curves of the Mn‐PAA and Mn‐PAA/G electrodes are also shown in Figure S25 (Supporting Information). Although they share the similar basic redox features with the Mn‐PAA⊂GS⊂G, the non‐resolved peaks either in the cathodic or anodic scan suggest their relatively poorer reaction kinetics. During the subsequent cycles, the CV curves of the Mn‐PAA⊂GS⊂G basically overlap, meaning a stable surface/structure state of electrode materials together with good reversibility after the initial SEI layer formation and activation process.

Figure 5b presents the galvanostatic discharge/charge profiles of Mn‐PAA⊂GS⊂G for the first three cycles at 0.1 A g^−1^ between 0.01 and 3.0 V. It can deliver the discharge/charge capacities of 1176/748 mAh g^−1^ in the first cycle, yielding an initial Coulombic efficiency (CE) of ≈63.6%, which is largely surpass the Mn‐PAA (≈26.3%; Figure S25c, Supporting Information) and Mn‐PAA/G (≈41.5%; Figure S25d, Supporting Information). It is worth mentioning that the bare PAA exhibits an almost irreversible storage property for Li^+^ ions, since it is only able to deliver a very poor initial CE of 10.1% in the first cycle together with ultralow discharge/charge capacities (109/11 mAh g^−1^; Figure S26, Supporting Information), which are then declined to ≈10 mAh g^−1^ in the following cycles, being consistent with our previously reported result.^[^ ^10^ ^]^ In addition, as compared with other two electrodes, the Mn‐PAA⊂GS⊂G electrode exhibits the smallest voltage hysteresis owing to the improved electrochemical reaction kinetics, which is in favor of improving the battery energy efficiency and reducing the risk of thermal runaway in high‐rate fast charging.

The rate capability was further investigated at various current densities ranging from 0.1 to 30 A g^−1^. As exhibited in Figure 5c, the Mn‐PAA⊂GS⊂G presents a considerably enhanced rate capability among all the samples at each current density. When the current density reaches 0.1, 0.2, 0.5, 1, 2, 5, 10, and 15 A g^−1^, the Mn‐PAA⊂GS⊂G delivers an average reversible capacity of 719, 636, 564, 512, 462, 391, 332, and 277 mAh g^−1^, respectively. More remarkably, even at the ultrahigh current rate of 20, 25, and 30 A g^−1^, the Mn‐PAA⊂GS⊂G electrode still exhibits a high reversible capacity of 226, 169, and 150 mAh g^−1^. Note that the high rate response at 30 A g^−1^ corresponds to an ultrafast charge and discharge time of ≈14 s. As a sharp contrast, the Mn‐PAA and Mn‐PAA/G only deliver negligible discharge capacities of ≈8 and 20 mAh g^−1^ at 30.0 A g^−1^, respectively. When the current reduced to 100 mA g^−1^, the Mn‐PAA⊂GS⊂G electrode could rapidly recover a reversible capacity of 745 mAh g^−1^, indicating its good structure stability. The excellent rate capability of the Mn‐PAA⊂GS⊂G is superior to its control samples as well as many other previously reported Sb–O based anode materials, as compared in Figure 5g.^[^ ^36^, ^52^, ^53^, ^54^, ^55^, ^56^, ^57^, ^58^, ^59^, ^60^, ^61^, ^62^, ^63^ ^]^ The specific capacity of the GS⊂G was also tested, as shown in Figure S26 (Supporting Information). It shows low reversible capacities (0.1 A g^−1^: 372 mAh g^−1^; 5 A g^−1^: 26 mAh g^−1^), indicating its limited capacity contribution. Meanwhile, highly stable CE values can be also obtained for the Mn‐PAA⊂GS⊂G electrode when cycling from low to high current density, much better than those for the Mn‐PAA and Mn‐PAA/G electrodes (Figure 5d), indicating the more stable surface state and structure of Mn‐PAA⊂GS⊂G that ensure the high Li^+^ storage reversibility.

To further validate the ability of the Mn‐PAA⊂GS⊂G composite to adapt ultrafast electronic and charge transport, rate testing at different charging and discharging models was carried out. Figure 5e shows the discharging rate performance of Mn‐PAA⊂GS⊂G, which was surveyed by charging a cell at a fixed current density of 0.1 A g^−1^ along with discharging from 0.1 to 30 A g^−1^, because the charging at 0.1 A g^−1^ is slow enough to charge the full capacity regardless of discharging rate. As clearly seen, the Mn‐PAA⊂GS⊂G electrode can deliver reversible discharge capacities over 500 mAh g^−1^ when the discharging rate ranging from 0.1 to 10 A g^−1^, while the discharge capacity can still retain beyond 200 mAh g^−1^ even as raising the discharging rate to 30 A g^−1^ (discharging time: ≈31 s). More specifically, the discharge capacity can respectively reach 535, 295, and 238 mAh g^−1^ at 10, 25, and 30 A g^−1^, indicating the ultrafast but high‐efficiency capacity release capability of the Mn‐PAA⊂GS⊂G electrode at the high rate discharging. Likewise, the charging rate capability of the cell was also evaluated by varying the charging current rate from 0.1 to 80.0 A g^−1^ while fixing the discharging current rate at 0.1 A g^−1^ (Figure 5f). When the charging rate was performed at 0.1, 0.2, 0.5, 1, 2, 5, and 10 A g^−1^, the charging capacity of Mn‐PAA⊂GS⊂G electrode can reach 681, 672, 653, 634, 610, 566, and 522 mAh g^−1^, respectively. As the charging current density was raised from 20 A g^−1^ to 30, 40, 50, 60 A g^−1^, the charge capacity of the tested electrode decreased slowly from 474 mAh g^−1^ to 448, 438, 431, and 425 mAh g^−1^, respectively. Even at an ultrahigh charging rate of 70 A g^−1^, it is able to deliver a reversible capacity of 390 mAh g^−1^, which is equal to ≈57% of the capacity at 0.1 A g^−1^. More impressively, a reversible capacity as high as 378 mAh g^−1^ can be still achieved, even if the charging rate has been elevated up to 80 A g^−1^, which is comparable to the theoretical capacity of graphite anode. In other words, the Mn‐PAA⊂GS⊂G can fill up 56% of its capacity within only ≈17 s charging time, suggesting its unprecedented superfast‐charging capability. As far as we know, this is the fastest charging speed together with a record charging efficiency to date among all the previously reported Sb–O based anode materials.

To further explicate the high rate capability, the CV experiments at different scan rates ranging from 0.1 to 5.0 mV s^−1^ were implemented to investigate the reaction kinetics of the Mn‐PAA⊂GS⊂G electrode (Figure S28a, Supporting Information). The CV curves of the Mn‐PAA⊂GS⊂G at different scan rates manifest similar shapes and comparable peak separations, reflecting a good transfer kinetics at high rates. According to recent studies, the charge stroage mechanism can be divided to two ways, diffusion‐controlled faradaic storage and surface‐controlled capacitive storage.^[^ ^64^, ^65^, ^66^ ^]^ The two storage process can be characterized by the following power law relationship between current (*i*) and scan rate (*v*), *i* = *av* ^b^. The *b* value can be calculated from the plots of log(i) versus log(*v*) and represents different storage mechanism (0.5: diffusion‐controlled faradaic storage; 0.5–1: hybrid strorage; 1: capacitive storage). As displayed in Figure S28b (Supporting Information), the *b* values were calculated to be 0.88 and 0.84 for cathodic (peak 1) and anodic (peak 2), respectively. This indicates the Li^+^ ions storage process of the Mn‐PAA⊂GS⊂G is dominantly controlled by the surface‐controlled capacitive process, thus giving rising to the high rate property.^[^ ^64^ ^]^ The capacitive storage contribution can be further qualified by distinguishing the current (*i*) into capacitive effects (*k*_1_ *v*) and diffusion‐controlled reactions (*k*_2_ *v* ^1/2^):

(1) $$\begin{array}{c} i\left(V\right)=k_{1}v+k_{2}v^{1/2} \end{array}$$

Figure S28c (Supporting Information) shows a typical capacitive storage contribution (blue region) in comparison to the total charge storage. A capacitive contribution of 68.6% can be obtained for the Mn‐PAA⊂GS⊂G electrode at 1 mV s^−1^. As the scan rate increases, the capacitive contribution boosts significantly, reaching a maximum value of 93% at 5 mV s^−1^ (Figure S28d, Supporting Information). The dominant capacitive contribution for the Mn‐PAA⊂GS⊂G stems from its unique hierarchical porous and multidimensional assembled 0D⊂1D⊂2D architecture, which can provide omnidirectional electronic transport network and more surface areas to access Li^+^ ions with faster charge transfer kinetics. This can be further reflected by the electrochemical impedance spectroscopy (EIS) analysis for the electrodes (Figure S29, Supporting Information). As summerized in Table S5 (Supporting Information), the charge transfer resistance (*R*_ct_) value of Mn‐PAA⊂GS⊂G electrode (81.7 Ω) is much smaller than that of the Mn‐PAA (235.6 Ω), and Mn‐PAA/G (150.5 Ω), highlighting the considerably enhanced kinetics of charge transportation provided by the structural advantages of Mn‐PAA⊂GS⊂G.^[^ ^67^ ^]^

Apart from the unprecedent rate capability, the multidimensional integrated architecture also renders the Mn‐PAA⊂GS⊂G composite with excellent long‐term cycling performance. Figure 5h presents the cycling performance of the three electrodes at a high current density of 1.0 A g^−1^ for 1000 cycles. The initial reversible capacity of the Mn‐PAA⊂GS⊂G electrode is ≈510 mAh g^−1^. Then, it gradually grows to 1283 mAh g^−1^ after 700 cycles and can discharge 1077 mAh g^−1^ even after 1000 cycles. Also, nearly 100% CE is observed during the overall cycling process except for the first cycle. As a contrast, the Mn‐PAA/G electrode is gradually decreased from 276 to 166 mAh g^−1^ after 1000 cycles, while the Mn‐PAA exhibits a rapid capacity loss in the first 100 cycles, followed by delivering only an ultralow capacity of 73 mAh g^−1^ after 1000 cycles. To our best knowledge, there is no any Sb–O based LIB anode material that can achieve such a high‐rate cycling performance comparable to the Mn‐PAA⊂GS⊂G composite developed in our work (Table S6, Supporting Information). There is an obvious capacity increase occurred for Mn‐PAA⊂GS⊂G electrode, which is common for Mn‐containing anode materials.^[^ ^68^, ^69^, ^70^ ^]^ This phenomenon can be explained by the following reasons: (i) The close wrapping of GS makes the embedded Mn‐PAA nanocrystals inadequately activated in the initial cycles and not fully contacted with the electrolyte. As the cycling goes further, more imbedded nanocrystals could be exposed and activated so as to persistently deliver capacity; (ii) The Mn^2+^ may be oxidized to higher valence states (Mn^3+^ and/or Mn^4+^) during the charge process, which can be reflected in the discharge/charge profiles and differential capacities versus voltage (dQ/dV) plots. As displayed in Figure S30a (Supporting Information), a voltage plateau and peak ≈2.5 V becomes increasingly obvious upon cycling, suggesting the oxidation conversion of Mn^2+^ to higher valence states (Mn^3+^ and/or Mn^4+^);^[^ ^31^, ^69^, ^71^ ^]^ (iii) the reversible reaction of a gel‐like polymer film, which can be verified by the intensified dQ/dV peak under 0.5 V (Figure S30b, Supporting Information).^[^ ^72^, ^73^ ^]^

**Figure 6:**
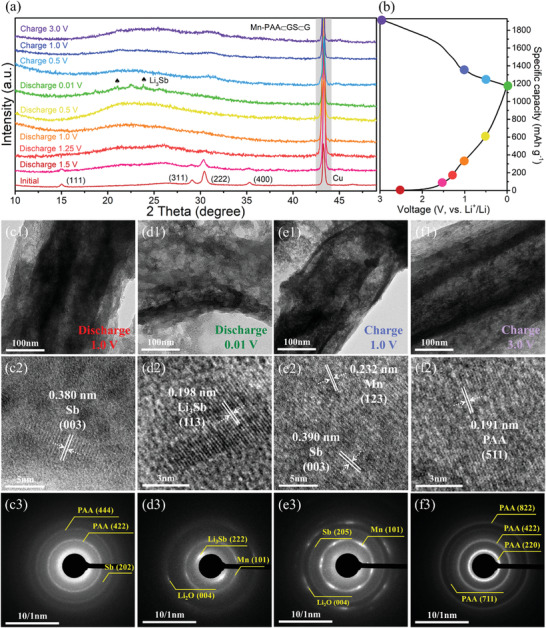
a) Ex situ XRD patterns of the Mn‐PAA⊂GS⊂G in the first cycle; b) First discharge/charge curves of the Mn‐PAA⊂GS⊂G; TEM images, HRTEM image, and SAED patterns of the Mn‐PAA⊂GS⊂G electrode after discharge to 1.0 V (c1–c3), 0.01 V (d1–d3) and charge to 1.0 V (e1–e3), 3.0 V (f1–f3) in the first cycle.

To disclose the electrochemical reaction mechanism, ex situ XRD and TEM were conducted to detect the Mn‐PAA⊂GS⊂G electrode (**Figure** **6**). As shown in Figure 6a, the diffraction patterns in the initial stage clearly shows the pyrochlore phase of the Mn‐PAA⊂GS⊂G with four typical diffraction peaks of (111), (311), (222), and (400), respectively, while the peak located at ≈43.2° can be assigned to the Cu collector. In the first discharge process from the initial stage to 1.0 V, the four main peaks are gradually shifting to lower angle, weakening and finally fading away, indicating the intercalation of Li^+^ ions and the occurrence of a conversion reaction, meanwhile the microstructure of the 1D Mn‐PAA⊂GS unit still remains unchanged (Figure 6c1). However, when the electrode discharged to 1.0 V, an interplanar distance of ≈0.380 nm can be observed in Figure 6c2, which is attributable to the formation of metallic Sb. Besides, the selected area electron diffraction (SAED) pattern (Figure 6c3) reveals the co‐existence of PAA phase and Sb metal. This verifies that part of the Mn‐substituted PAA was involved in the conversion reaction. When the electrode is discharged to 0.01 V, two new diffraction peaks (labeled by spade) appear and can be assigned to Li_3_Sb (Figure 6a), which is associated with the alloying reaction of Sb and Li. In the HRTEM image (Figure 6d2), an interplanar distance of ≈0.198 nm well corresponds to the (113) plane of Li_3_Sb, indicating that the metallic Sb generated by the conversion reaction is able to continually react with Li through an alloying reaction. In addition of the formation of Li_3_Sb, the simultaneous existence of Li_2_O and metallic Mn derived from the conversion reaction of Mn‐PAA was also evidenced by the SAED pattern (Figure 6d3). In the following charge process, the diffraction peaks of the Li_3_Sb disappeared and no any other peaks can be detected, demonstrating that the reaction products are amorphous or in a low crystalline state. From the HRTEM and SAED results of the electrode after charging to 1.0 V, (Figure 6e2,e3), however, a dealloying reaction can be verified by the recurrence of Sb, while there is no oxidation conversion reaction detected at this stage. This is well consistent with the abovementioned CV results. When fully charging back to 3.0 V (Figure 6f1), the HRTEM result (Figure 6f2) and SAED pattern (Figure 6f3) reveals the existence of pyrochlore phase PAA, verifying that sequential decomposition of Li_2_O and complete oxidation conversion of the Sb and Mn occurred.

Figure S31 (Supporting Information) exhibited the thickness of all the sample electrodes. For the Mn‐PAA electrode, after discharging to 0.01 V, it shows a large increase in its thickness (20.7 µm → 53.4 µm) and large volume expansion ratio (158%). After fully charged, the thickness of the Mn‐PAA electrode remained at 42.7 µm. For the Mn‐PAA/G electrode, it increases from 23.3 to 43.6 µm after discharging with a volume expansion ratio of about 87%. After charging to 3.0 V, the Mn‐PAA/G electrode can reduce to 32.3 µm. As for the Mn‐PAA⊂GS⊂G electrode, it shows a similar thickness (23.8 µm) with the Mn‐PAA/G electrode in the fresh state. However, it only increases to 27.5 µm after discharging, only corresponding to 16% volume expansion ratio. After charging to 3.0 V, the Mn‐PAA⊂GS⊂G electrode can back to 24.6 µm. This indicates an effective confinement of the multidimensional structure and good reversibility, which is in favor of its cycling performance.

To illuminate the impressive cycling performance of the Mn‐PAA⊂GS⊂G, postmortem characterizations were also carried out. Figure S32 (Supporting Information) compares the morphology variation of the Mn‐PAA⊂GS⊂G electrode before and after 100 cycles at 1.0 A g^−1^. Owing to the SEI formation on the surface of the Mn‐PAA⊂GS⊂G, the graphene becomes smooth and opaque. However, the integrated structure of the Mn‐PAA⊂GS⊂G is clearly visible and the typical 1D tubular Mn‐PAA⊂GS structure unit still remains as integrity without any crack, demonstrating its robust structural features. In addition to fresh state, the Mn‐PAA, Mn‐PAA/G, and Mn‐PAA⊂GS⊂G electrodes has been analyzed by ex situ EIS technique at 20th, 200th, and 500th cycles (Figure S33 and Table S7, Supporting Information). All the electrodes show a decrease of total resistance (*R*_all_), indicating an activation process and improved reaction kinetics after 20 cycles.^[^ ^74^ ^]^ However, for the Mn‐PAA and Mn‐PAA/G electrode, the *R*_all_ gradually increases with cycling, suggesting an unstable interface and continuing growth of SEI layer.^[^ ^74^, ^75^ ^]^ As a contrast, the Mn‐PAA⊂GS⊂G electrode shows a complete different situation. The *R*_all_ presents a stable total resistance (about 15 Ω) in the 200th and 500th cycles, reflecting its stable structure.

**Figure 7:**
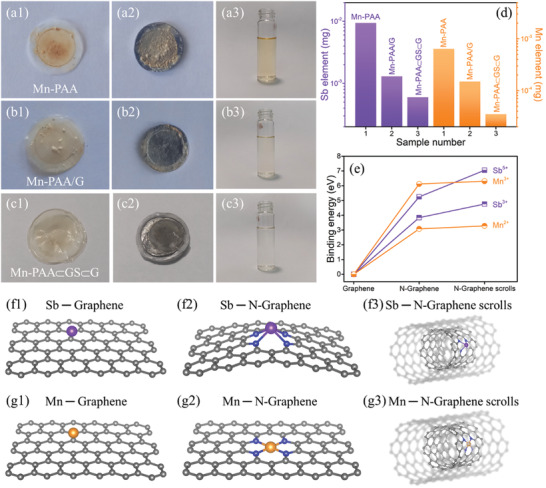
Photographs of membrane, Li electrode, and dissolved aqueous of Li electrode for the a1–a3) Mn‐PAA, b1–b3) Mn‐PAA/G, and c1–c3) Mn‐PAA⊂GS⊂G electrode after 1000 cycles; d) The mass of the Sb and Mn elements deposited on the Li counter electrode for the three samples; e) The binding energy results between metal ions and different graphene structures; f1–g3) Six models between two type metal ions and three type graphene.

After 1000 cycles, the cells with the Mn‐PAA, Mn‐PAA/G, and Mn‐PAA⊂GS⊂G electrodes were disassembled for detailed observation further. As for the Mn‐PAA cell, both the membrane and the Li electrode have become very dark yellow as their surface have been covered with something, as shown in **Figure** **7**a1,a2. Similarly, the cell with Mn‐PAA/G electrode also has the contaminated membrane and Li foil but exhibits a relative low degree (Figure 7b1,b2). In sharp contrast, the color change for either the membrane or the Li foil is not obvious in Mn‐PAA⊂GS⊂G‐adopted cell (Figure 7c1,c2). According to literatures,^[^ ^76^, ^77^, ^78^ ^]^ some metal ions are easily dissolved into the electrolyte and then deposited on the surface of the membrane and the Li counter electrode during long‐term cycling, which may be responsible for the color change observed in the cycled cells. Therefore, we further tested the contents of possible metal species deposited on the Li electrode by ICP‐OES after dissolving the cycled Li electrode in water. Obviously, the color of the dissolved solution for the three samples is very different, as compared in Figure 7a3–c3, in which the lightest color is witnessed for the Mn‐PAA⊂GS⊂G, suggesting that it may contains minimal soluble metal ions. As verified by the ICP‐OES results (Figure 7d), the Mn‐PAA⊂GS⊂G indeed has the smallest deposition of Sb (6.0 × 10^−4^ mg) and Mn (3.5 × 10^−5^ mg) element, indicating that there could be a positive effect of restraining the dissolution of active species from the Mn‐PAA⊂GS⊂G electrode materials.

To better understand the underlying inhibition effect, the DFT calculations were adopted to obtain the interaction information between graphene and Sb/Mn species. Based on the experimental results, six models were constructed, which comprise three types of graphene (i.e., graphene, N‐doped graphene, and N‐doped graphene scrolls) and two types of metal species (i.e., Mn and Sb), as shown in Figure 7f1–g3. Binding energy information for all the models is concluded in Figure 7e. Perfect graphene sheet has no interaction with the Mn^2+^/Mn^3+^ and Sb^3+^/Sb^5+^ ions, but the introduction of N into the graphene can notably enhance the interaction between graphene and metal ions with high binding energy, such as Mn^2+^ (3.08 eV), Mn^3+^ (6.12 eV), Sb^3+^ (3.84 eV), and Sb^5+^ (5.25 eV). Moreover, due to the unique spatially geometric configuration, N‐doped 1D graphene scrolls are predicted to exhibit a relatively higher binding energy for these metal ions including Mn^2+^(3.28 eV), Mn^3+^(6.31 eV), Sb^3+^(4.76 eV), and Sb^5+^ (7.05 eV), as compared with the N‐doped 2D graphene sheet. In addition to the chemical adsorption effect endowed by the doped N heteroatom, multilayer tubule‐shaped graphene scrolls can also act as a physical barrier to further prevent the loss of active Sb and Mn species, as illustrated in Figure 7f1,g3. Informed by these DFT calculations, it is effective for the Mn‐PAA⊂GS⊂G architecture to maximally relieve the dissolution of active Mn and Sb species as a result of the structural advantages of N‐heteroatom doping and geometric confinement, thereby being in favor of improving the structural integrity and cycling stability of electrode materials.

Based on the above results, the remarkably enhanced Li^+^ ions storage reversibility of the Mn‐PAA⊂GS⊂G composite together with ultrahigh rate capacity, superfast charging capability, and long cycling life are mainly ascribed to the following advantages. (i) The substitution of Mn^2+^ into the pyrochlore structure of PAA not only leads to a considerably narrowed bandgap but also greatly reduces the migration energy barrier of Li^+^ ions in the open tunnels, thus synchronously boosting the inherent electronic and ionic conductivity of PAA; (ii) 0D Mn‐PAA nanocrystals have high reaction activity and short ion transport distance, whilst the high conductive 1D GS along with 2D graphene sheets can provide rapid bicontinuous electron and ion transport channels, guaranteeing the ability of the composite to adapt the extremely fast charge transfer; (iii) the 3D architecture assembled from the 0D⊂1D⊂2D structure provides a stable buffer framework, meanwhile the abundant internal void can accommodate the volume expansion of the embedded Mn‐PAA, preventing the capacity fade upon cycling by keeping the electrode structure intact; (iv) the trapping from the N‐heteroatom together with the geometric confinement by the GS give rise to a strong chemical/physical interaction for immobilizing Sb and Mn species, thereby effectively relieving their loss from the electrode materials during long‐term cycling.

## 3 Conclusion

In summary, a multidimensional integrated architecture, which consists of 0D Mn‐substituted PAA⊂1D graphene scrolls⊂2D graphene sheets, was constructed based on a hydrothermally assisted smart strategy. We have demonstrated, for the first time, that the substitution of Mn^2+^ into the tunnel sites of the pyrochlore‐phase PAA can effectively improve its inherent bulk electronic conductivity by narrowing the bandgap and reducing the Li^+^ migration energy barriers, which is very favorable for enhancing the electrochemical reversibility of PAA with fast reaction kinetics. Meanwhile, the multidimensional assembled architecture combined with advantages of 0D, 1D, and 2D subunit builds up an omnidirectional electron/ion transport network and robust 3D composite structure. Experimental results along with theoretical calculations prove that the above merits cooperatively enable improved reaction kinetics, adaptive volume expansion, and relieved dissolution of active species, which allow the developed Mn‐PAA⊂GS⊂G anode exhibits remarkable electrochemical performance including ultrahigh rate capacity, superfast‐charging capability, and long cycling life. More importantly, the facile and efficient element substitution method developed in our work could be also extended to design and fabricate other metal species (e.g., Na^+^, Ca^2+^, Zn^2+^, Sn^2+^, Bi^3+^)‐substituted tunnel‐type PAA electrode materials with delicately modulated physicochemical properties toward other functional energy storage applications.

## Conflict of Interest

The authors declare no conflict of interest.

## Supporting information

Supporting Information
