# Supplementary material for: Mn‐Substituted Tunnel‐Type Polyantimonic Acid Confined in a Multidimensional Integrated Architecture Enabling Superfast‐Charging Lithium‐Ion Battery Anodes
Source: Adv Sci (Weinh). 2020 Nov 25;8(3):2002866. doi: 10.1002/advs.202002866 (PMC7856895; doi:10.1002/advs.202002866)
Supplement: Supplementary file 1 — Supporting Information [file ADVS-8-2002866-s001.pdf]

WILEY-VCH

© 2020 Wiley-VCH GmbH

ADVANCED SCIENCE
Open Access

Supporting Information

for *Adv. Sci.*, DOI: 10.1002/advs.202002866

Mn-Substituted Tunnel-Type Polyantimonic Acid Confined in a Multidimensional Integrated Architecture Enabling Superfast-Charging Lithium-Ion Battery Anodes

Boya Wang, Yunhong Wei, Haoyu Fang, Xiaoling Qiu, Qiaobao Zhang, Hao Wu, Qian Wang, Yun Zhang* and Xiaobo Ji*

Supporting Information

Mn-Substituted Tunnel-Type Polyantimonic Acid Confined in a Multidimensional Integrated Architecture Enabling Superfast-Charging Lithium-Ion Battery Anodes

Wang, Yunhong Wei, Haoyu Fang, Xiaoling Qiu, Qiaobao Zhang, Hao Wu, Qian Wang, Yun Zhang* and Xiaobo Ji*

Experimental Section

Chemical and reagents: All chemicals were purchased from commercial sources and used without purification. $KSb(OH)_6$ (CAS No. 12208-13-8) was purchased from Klamar Reagent Company, China. Poly(diallyldimethylammonium chloride) (PDDA) was purchased from Shanghai Aladdin Bio-Chem Technology Co., Ltd, Shanghai, China. Graphene oxide (GO) was purchased from Suzhou Tanfeng Graphene Technology Co., Ltd, Suzhou, China. Other reagents were obtained from Kelong Chemical Reagent Plant, Chengdu, China.

Synthesis of MnO_2 nanowires: Uniform MnO_2 nanowires were synthesized by a hydrothermal method as reported previously[1]. In a typical synthesis process, 7 mmol $KClO_3$, 4.0 mmol $MnSO_4{\cdot}H_2O$, and 7 mmol CH_3COOK were successively dissolved in 60 mL H_2O. Then, 3.2 mL CH_3COOH was added into the above aqueous solution under magnetic stirring. Subsequently, the mixed solution was transferred into a 100 mL Teflon-lined autoclave and maintained at 160 °C for 12 h. Finally, brown MnO_2 nanowires were obtained after filtration, washing, and drying.

Synthesis of MnO_2@Mn-PAA composite nanowires: Firstly, 200 mg MnO_2 nanowires were

dispersed in 100 mL PDDA aqueous solution (1 wt% PDDA). After sonification for 1 h, the MnO_2 slurry was thoroughly washed by centrifugation to remove superfluous PDDA. Then, 160 mg $KSb(OH)_6$, 900 mg urea, and 37 mL H_2O were added into the PDDA-modified MnO_2 to form a homogeneous slurry. Next, a water bath was used to dissolve the $KSb(OH)_6$ by heating the temperature to 85 °C. After adding into 23 mL CH_3OH, the mixed solution was transferred to a 100 mL Teflon-lined autoclave. After hydrothermal reaction at 170 °C for 12 h, filtration, and washing, brown-colored products of MnO_2@Mn-PAA composite nanowires were obtained finally.

Synthesis of Mn-PAA⊂GS⊂G: Typically, the obtained MnO_2@Mn-PAA (200 mg) was added into 100 mL of 1 wt% PDDA aqueous solution and ultrasonically treated for 1 h to form a uniform dispersion. The excess PDDA was then removed by repeated centrifugation/washing/redispersion cycles. The obtained aqueous dispersion of PDDA-modified MnO_2@Mn-PAA was further diluted to 100 mL, followed by the dropwise addition of 40 mL GO (0.75 mg mL^{-1}) aqueous solution under magnetic stirring for 30 min to drive an electrostatic attraction between the positively charged PDDA-modified MnO_2@Mn-PAA nanowires and the negatively charged GO sheets. After that, the MnO_2@Mn-PAA/GO was collected through centrifugation, washing, and freeze-drying.

The as-prepared MnO_2@Mn-PAA/GO was then homogeneously dispersed in 26 mL of urea (70 mg mL^{-1}) aqueous solution, followed by the addition of 4 mL of GO aqueous dispersion (7.5 mg mL^{-1}), and sealed in a 50 ml Teflon-lined autoclave. After hydrothermal treatment at 120 °C for 3 h, the autoclave was naturally cooled to room temperature, and the as-formed 3D macroscopic hydrogel was taken out and freeze dried, followed by annealing at

400 °C for 2 h in argon atmosphere to generate MnO_2@Mn-PAA confined in a multidimensional integrated architecture (MnO_2@Mn-PAA⊂GS⊂G). Then, the annealed product was dispersed in 0.5 M oxalic acid solution for 5 h to remove the MnO_2 core, and finally obtain the 3D Mn-PAA⊂GS⊂G composite after washing and drying. In addition, reduced GO was also prepared through the abovementioned hydrothermal treatment and annealing process.

Synthesis of Mn-PAA and Mn-PAA/G: The Mn-PAA was directly obtained by etching the MnO_2@Mn-PAA composite nanowires using 0.5 M oxalic acid solution. The Mn-PAA/G was prepared by mechanically mixing the the above-obtained bare reduced GO and Mn-PAA.

Materials Characterization: The crystalline phases of the obtained product were identified by X-ray diffraction (XRD, Philips X'pert TROMPD, Cu Kα1 radiation, λ=1.54178 Å). The Raman spectra were recorded on a Raman spectrophotometer (Horiba Jobin Yvon, HR800, France) with 532.17 nm laser radiation in the range of 100-2000 cm^{-1}. The morphology and microstructure of the sample were examined by a field emission scanning electron microscopy (FESEM, Hitachi, S-4800, Japan) and a field emission transmission electron microscopy (TEM, FEI, Titan themis 200, USA). Nitrogen adsorption/desorption isotherms and pore size distribution were characterized by Kubo-X1000 analyzer (Beijing Builder Electronic Technology Co., Ltd). The surface area was calculated by the Brunauer-Emmett-Teller (BET) method. And the pore size distributions were derived from the desorption branches of isotherms using the Barrett-Joyner-Halenda (BJH) model. The total pore volume was calculated at a relative pressure of 0.99 (P/P_o). The content of C and N was determined by an Elemental Analyzer (Leeman Labs, Euro EA 3000). The ultraviolet visible

(UV-vis) adsorption spectrum and UV-vis diffused reflectance spectrum (UV-vis DRS) were tested by an UV-vis spectrometer (Shimadzu, UV3600, Japan). The contents of metal ions were tested by inductively coupled plasma optical emission spectroscopy (ICP-OES).

Electrochemical measurements: To evaluate the electrochemical performance of the product as anode for lithium ion batteries, CR2032 coin-type cells were assembled in an argon-filled glove box using lithium foil as counter electrode and Celgard 2400 as separator, respectively. The anode slurry was prepared by mixing 70 wt% active materials, 20 wt% acetylene black and 10 wt% carboxymethyl cellulose (CMC) in deionized water, and then coated on Cu foil dried at 80 °C for 12 h. The mass of the active materials was kept approximately at ≈1.5 mg cm^{-2}. The final sheet was punched into disc (Φ = 12 mm) as the working electrode. The electrolyte is consisted of a solution of 1.0 M $LiPF_6$ solution in a mixture of ethylene carbonate, ethylmethyl carbonate, and dimethyl carbonate (EC/DMC/DEC, 1: 1: 1 vol%) with 5% fluoroethylene Carbonate (FEC). The discharge and charge performance were determined by an automatic Neware battery cycler (CT-4008T-5V-20/50mA, Shenzhen, China) in a voltage range of 0.01-3 V at room temperature. Cyclic voltammetry (CV) were conducted by a PARSTAT multichannel electrochemical workstation (Princeton Applied Research, PMC1000DC, USA) between 0.01 and 3 V (*vs.* Li/Li^+). Electrochemical impedance spectra (EIS) were performed by a PARSTAT electrochemical workstation (Princeton Applied Research, PMC1000, USA), using an AC voltage of 5 mV amplitude in the frequency range from 100 KHz to 0.1 Hz.

Density Functional Theory Calculations: The density functional theory (DFT) calculations were performed to calculate density of states in materials. The projected augmented wave

(PAW) method was implemented to calculated the ion-electron interactions. The exchange-correlation energy of electrons is treated with the PBE function. The energy cutoff is up to 400 eV, and a criterion of 10^{-4} eV/atom was set for the self-consistent convergence. In addition, for the Sb and Mn atoms, the U schemes need to be applied, and the U has been set as 1.5 and 3.2 eV. The lattice constant and atom position are fully relaxed. Li ions migration barrier energies had been evaluated using the climbing nudged elastic band (CI-NEB) methods. As for the adsorption energy, DFT calculations were performed with the Amsterdam Density Functional program package (ADF)1-2. The exchange-correlation energy between electrons were treated with the Becke (exchange) and the Lee-Yang-Parr (correlation) functional (BLYP)3. A double-ζ plus polarization basis (DZP) set is employed for all elements. The criterion of the self-consistent convergence of the total energy is set as 0.001 eV/atom. Due to the fact that antimony and cobalt are transition metals, the relativistic effect is considered.

Supporting Figures and Tables

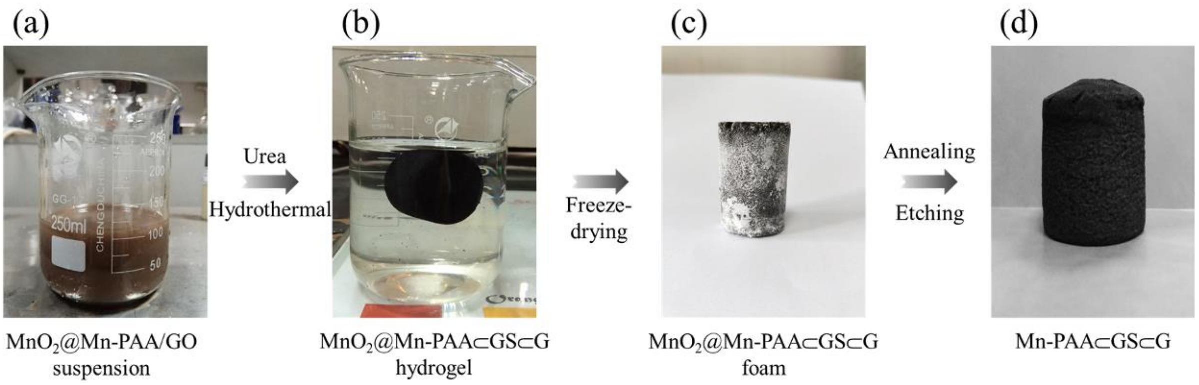

Figure S1 The typical digital photographs for illustrating the synthetic procedure of the Mn-PAA⊂GS⊂G.

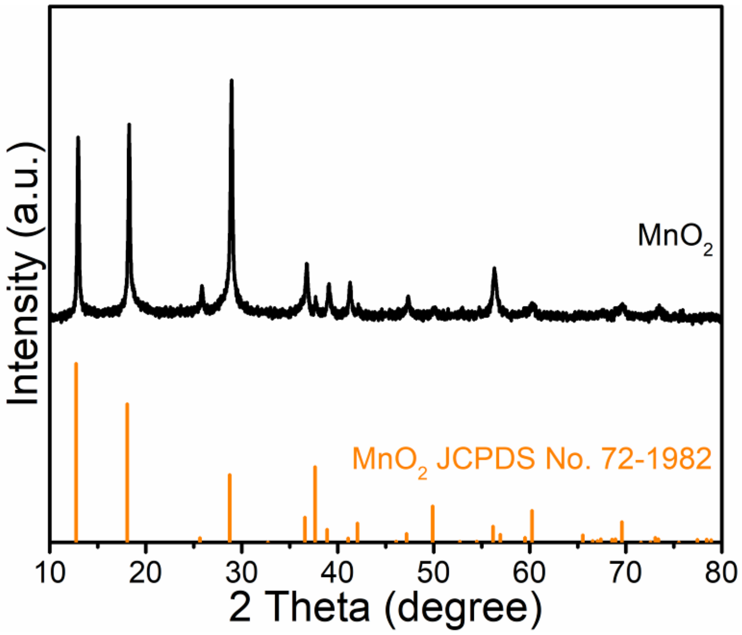

Figure S2 XRD pattern of the MnO_2 nanowires.

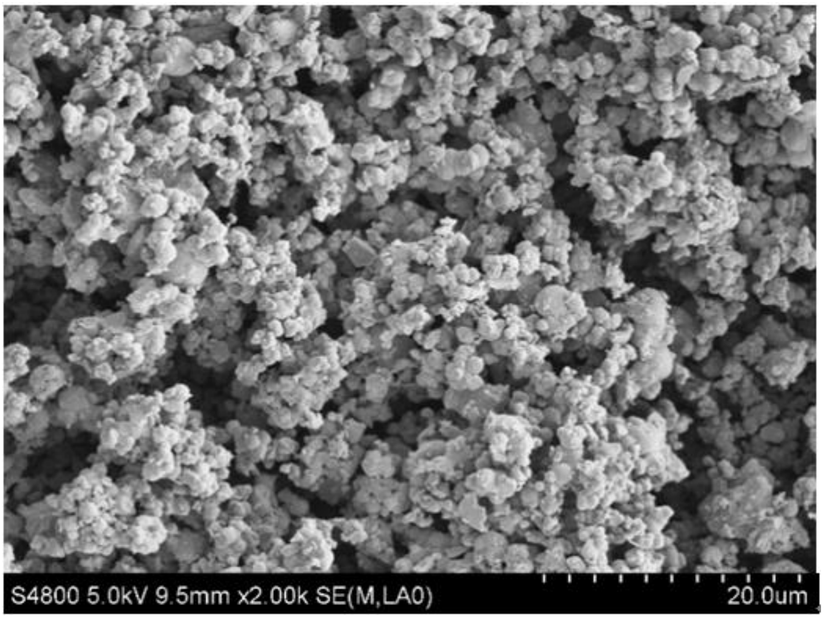

Figure S3 SEM images of the $KSb(OH)_6$.

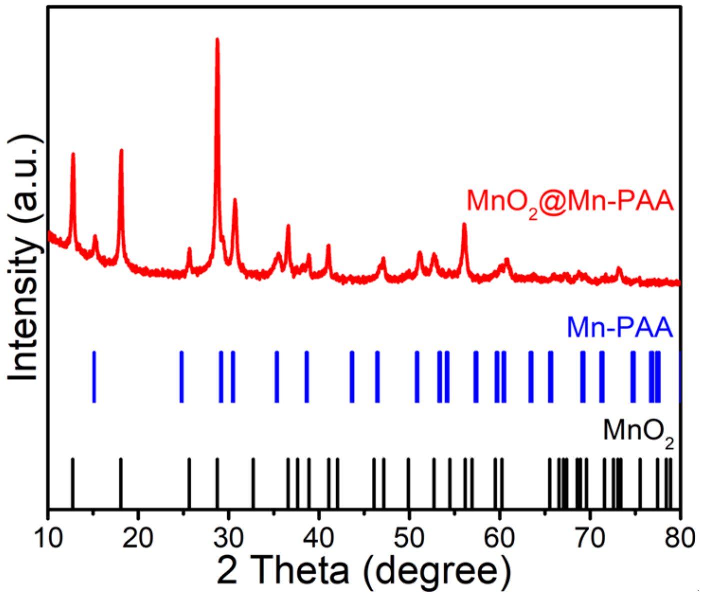

Figure S4 XRD pattern of the MnO_2@Mn-PAA.

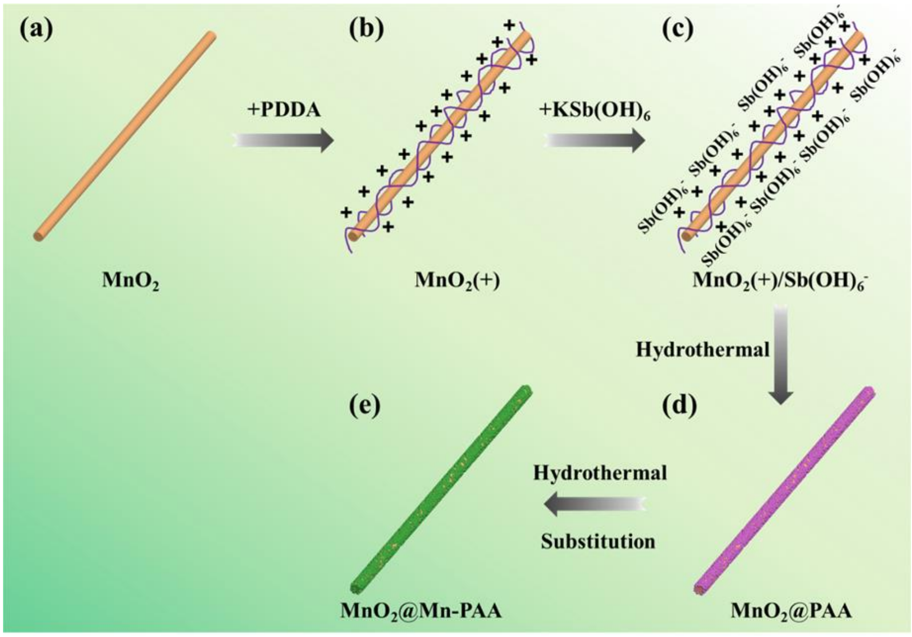

Figure S5 The synthesis mechanism of the MnO_2@Mn-PAA.

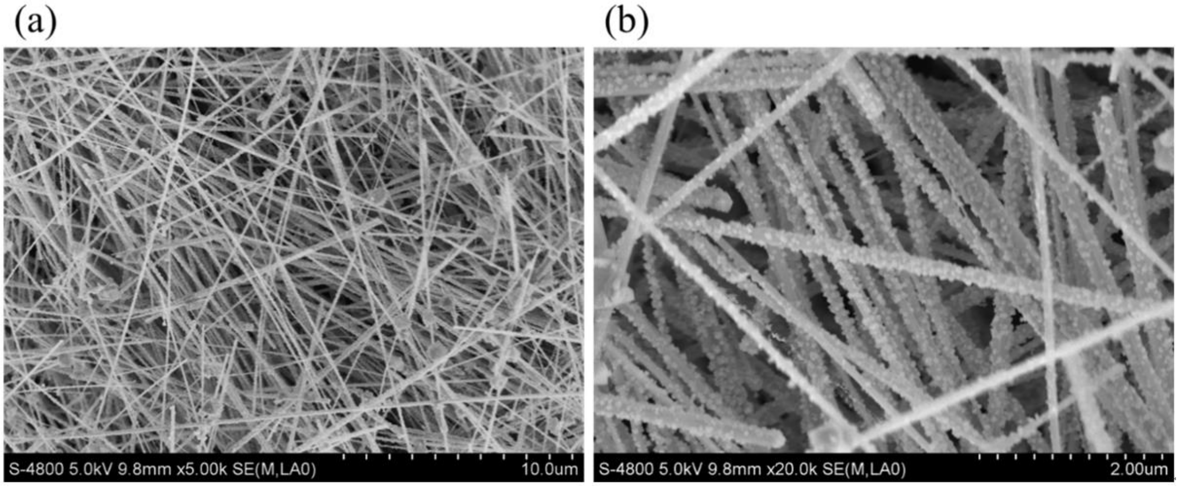

Figure S6 FESEM images of the MnO_2@Mn-PAA without the employment of PDDA.

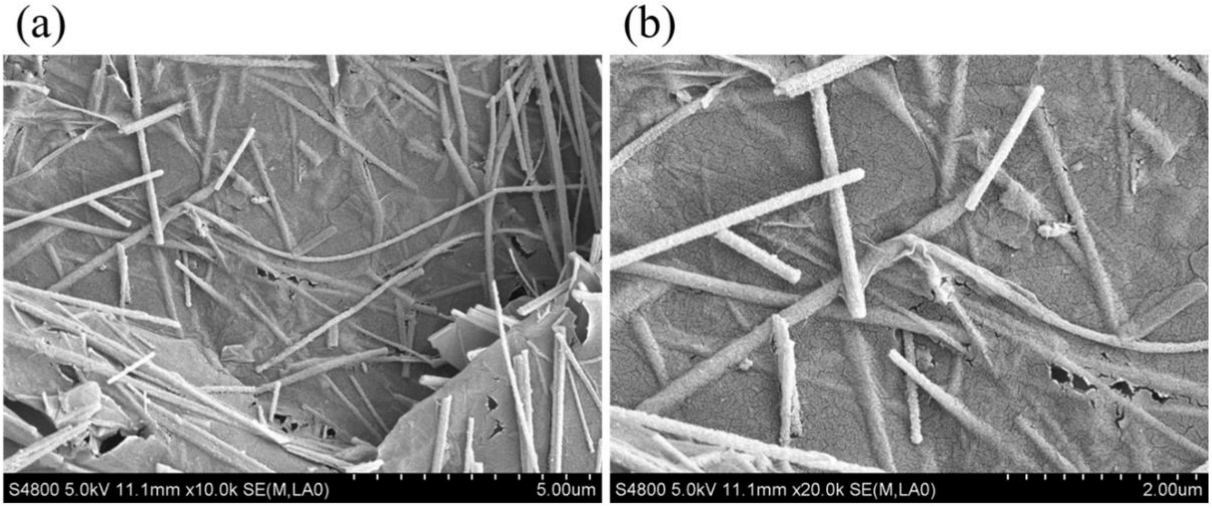

Figure S7 FESEM images of the MnO_2@Mn-PAA/GO.

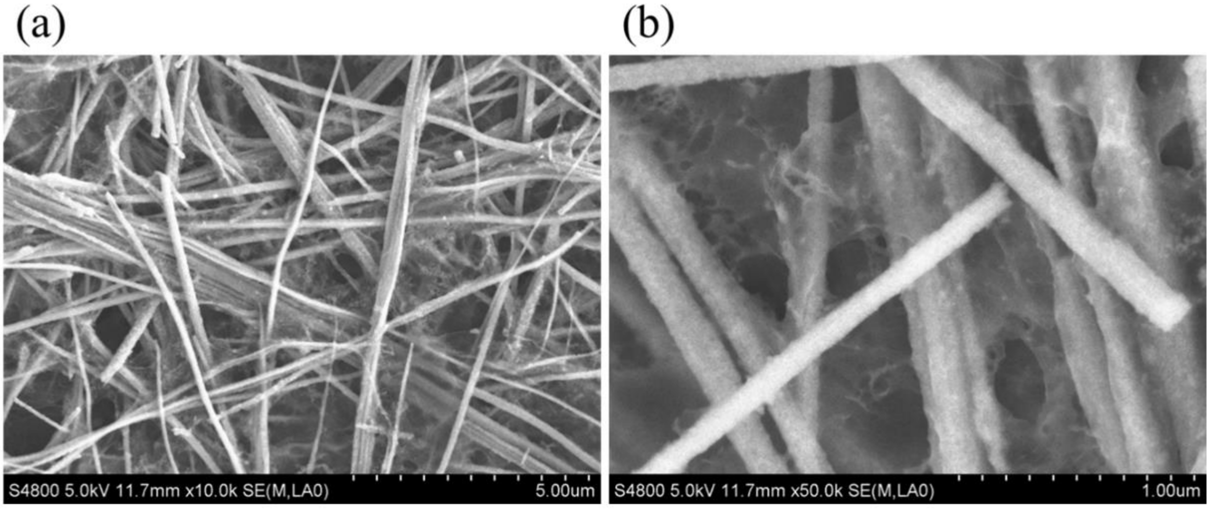

Figure S8 FESEM images of the MnO_2@Mn-PAA⊂GS⊂G.

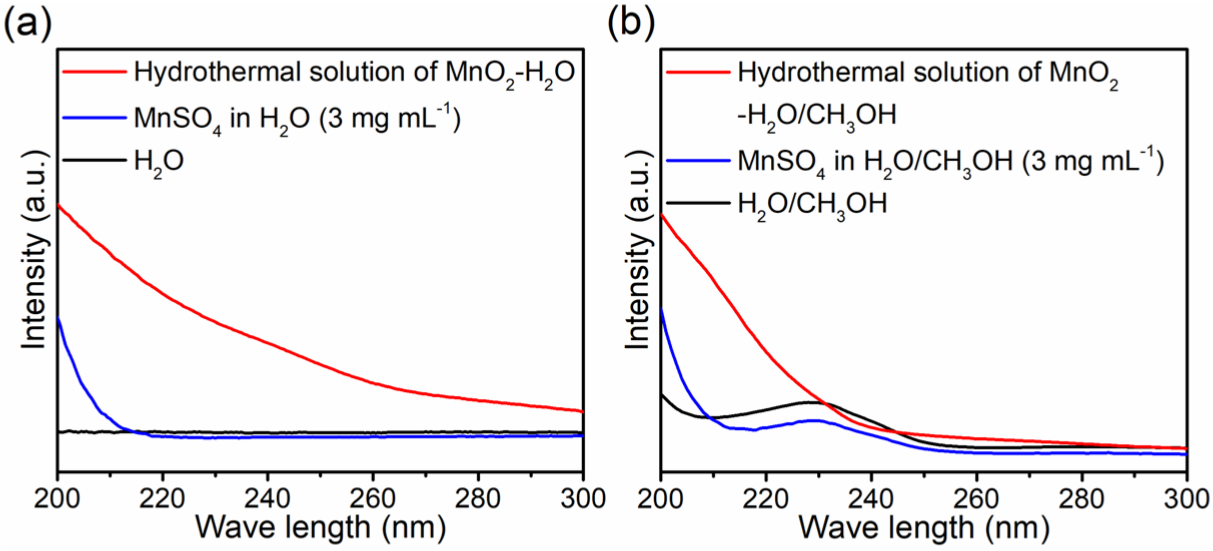

Figure S9 UV-vis spectra of different samples.

As shown in Figure S9a, there is no any absorption feature for H_2O solution. As for the hydrothermal solution containing MnO_2 and H_2O, it has an absorption edge near the 200 nm, which is similar with the $MnSO_4$ aqueous solution (3 mg mL^{-1}), suggesting the existence of Mn^{2+} ions. In Figure S9b, the H_2O/CH_3OH mixed solution has an absorption peak (~230 nm) and an absorption edge near the 200 nm. The $MnSO_4$ solution (3 mg mL^{-1}) with H_2O/CH_3OH solvent has similar characteristics with H_2O/CH_3OH solvent but has an enhanced intensity at the absorption edge near the 200 nm originating from Mn^{2+} ions. The hydrothermal solution of containing MnO_2-H_2O/CH_3OH also indicates an increased absorption edge compared with the $MnSO_4$ solution, which also verifies the generation of Mn^{2+} ions. These results are also accordance with fact that manganese ion mainly exists with bivalence in the neutral, acidic, or reductive environments.

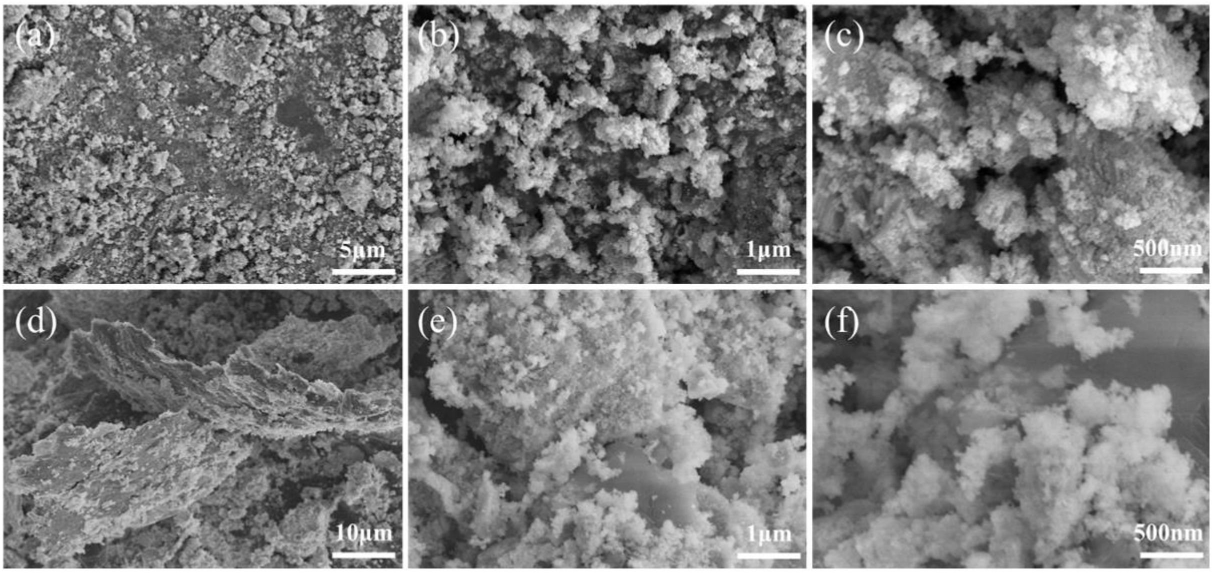

Figure S10 FESEM images of the (a-c) Mn-PAA and (d-f) Mn-PAA/G.

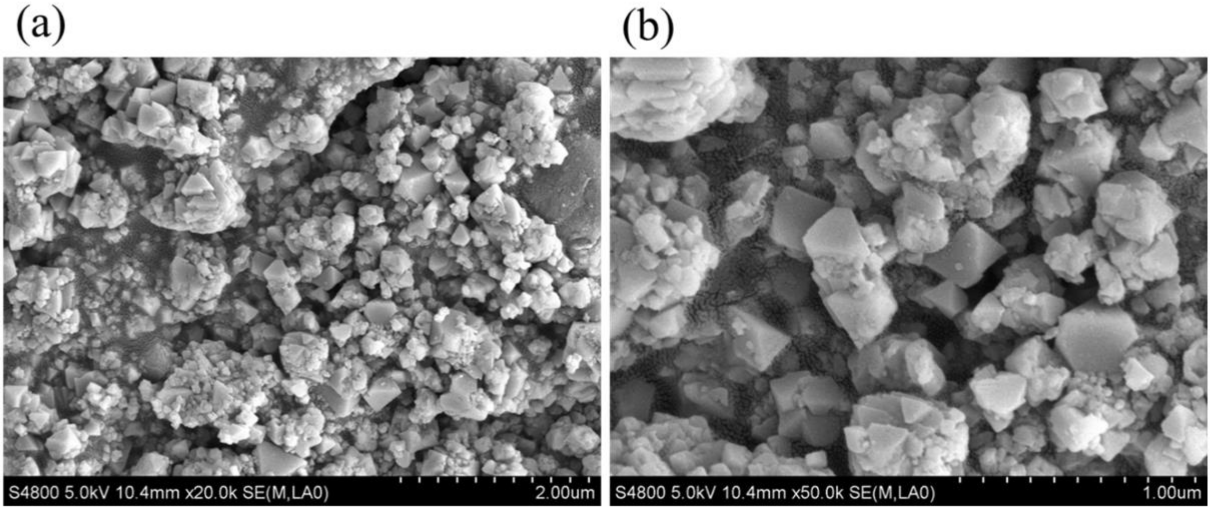

Figure S11 FESEM images of the bare PAA.

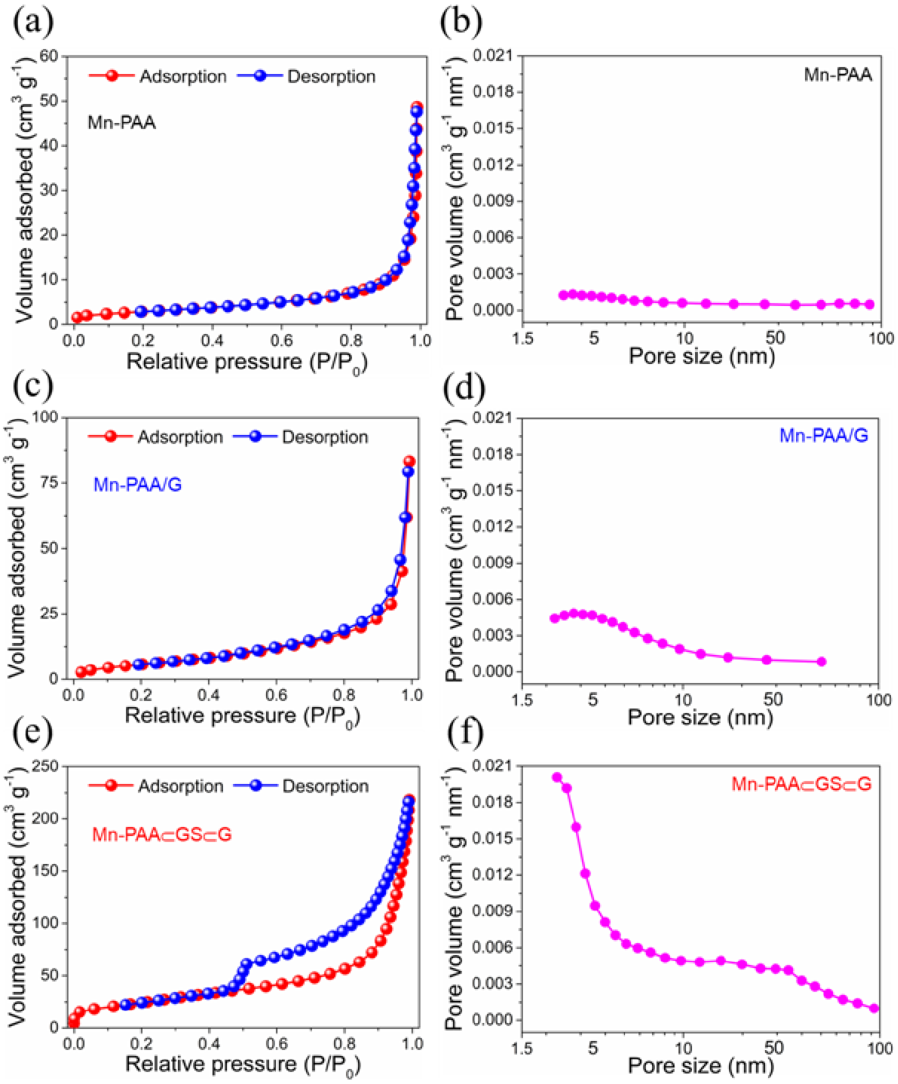

Figure S12 N_2 adsorption/desorption isotherms and pore-size distribution plots as calculated by the BJH method from the adsorption branch isotherm for the (a, b) Mn-PAA, (c, d) Mn-PAA/G, and (e, f) Mn-PAA⊂GS⊂G.

Table S1 BET specific surface area and the porosity of the Mn-PAA, Mn-PAA/G, and Mn-PAA⊂GS⊂G samples.

Samples	BET specific surface area (m^2 g^{-1})	BJH pore volume (cm^3 g^{-1})
Mn-PAA	10.6	0.076
Mn-PAA/G	22.2	0.133
Mn-PAA⊂GS⊂G	90.1	0.341

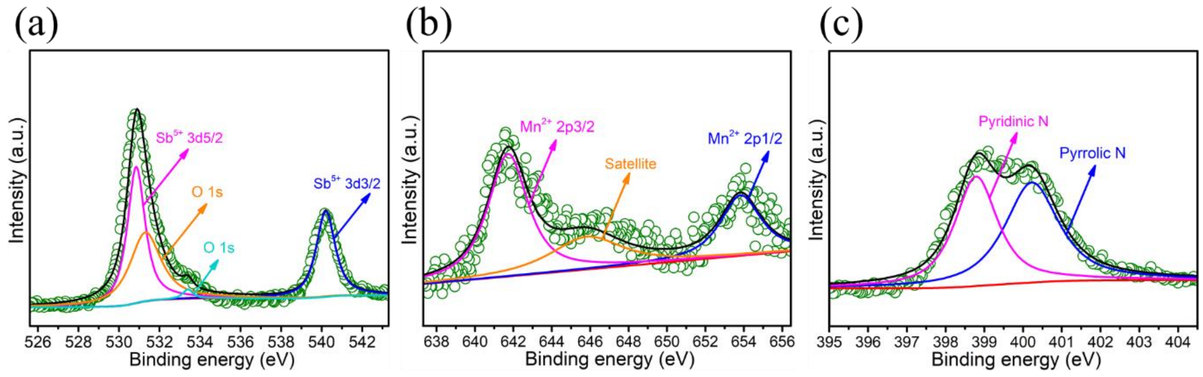

Figure S13 (a) Sb 3d and O 1s spectrum of the Mn-PAA⊂GS⊂G; (b) Mn 2p and (c) N 1s spectra of the Mn-PAA⊂GS⊂G.

Table S2 The content of C and N elements in the Mn-PAA/G and Mn-PAA⊂GS⊂G based on EA analysis.

Sample	C (wt%)	N (wt%)	Total
Mn-PAA/G	40.2	5.7	45.9
Mn-PAA⊂GS⊂G	35.5	9.9	45.4

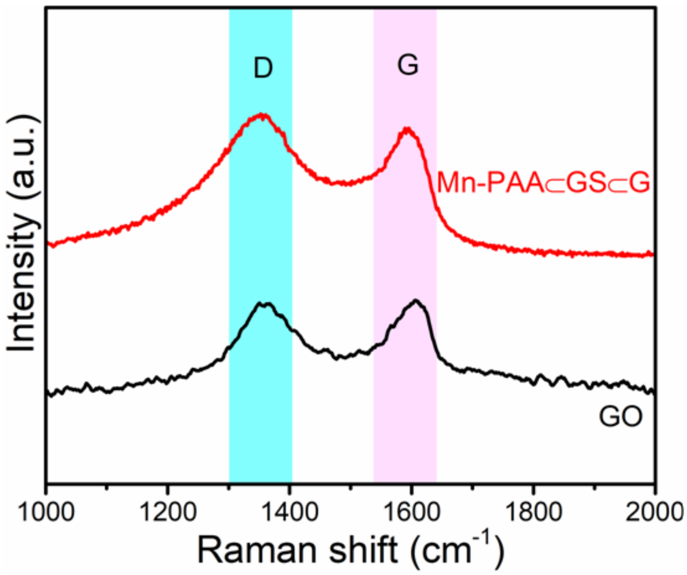

Figure S14 Raman spectra of the GO and Mn-PAA⊂GS⊂G.

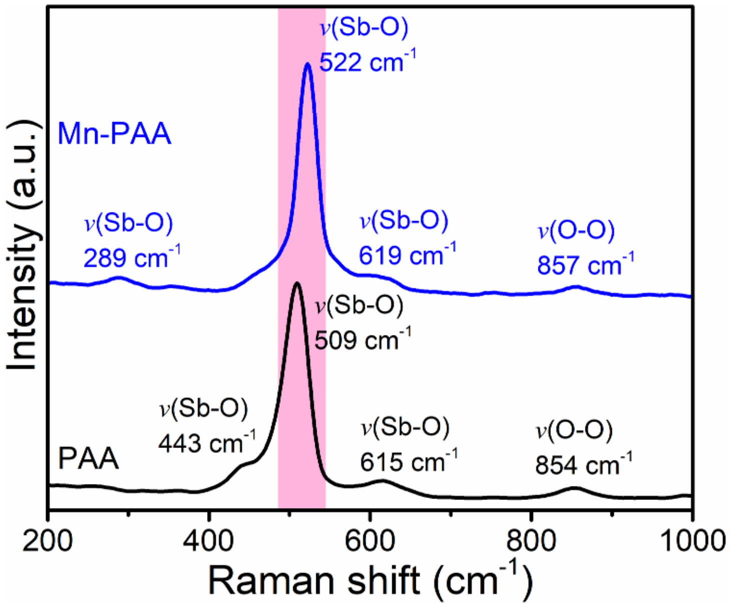

Figure S15 Raman spectra of the bare PAA and Mn-PAA.

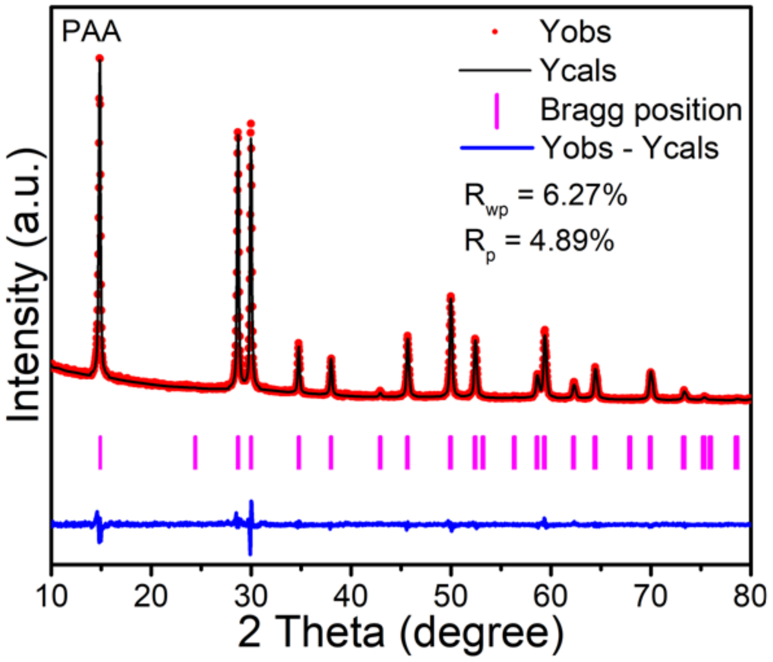

Figure S16 The Rietveld refinement patterns of the bare PAA.

Table S3 Results of structural analysis obtained from XRD Rietveld refinement of the bare PAA and Mn-PAA⊂GS⊂G.

**Samples**	**Reference PAA (COD ID 1529908)**	**PAA**	**Mn-PAA⊂GS⊂G**
Space group	$Fd\bar{3}m$	$Fd\bar{3}m$	$Fd\bar{3}m$
$a(=b=c)$ (Å)	10.363	10.333	10.162
$\alpha(=\beta=\gamma)$ (°)	90	90	90
Z	8	8	8
V (Å^3)	1112.9	1103.3	1049.4
16c	Sb (0, 0, 0)	Sb (0, 0, 0)	Sb (0, 0, 0)
48f	O1 (0.332, 0.125, 0.125)	O1 (0.322, 0.125, 0.125)	O1 (0.354, 0.125, 0.125)
8b	O2 (0.375, 0.375, 0.375)	O2 (0.375, 0.375, 0.375)	O2 (0.375, 0.375, 0.375)
16d	O3 (0.5, 0.5, 0.5)	O3 (0.5, 0.5, 0.5)	Mn (0.5, 0.5, 0.5)
R_{wp} (%)	/	6.27	4.56
R_p (%)	/	4.89	3.34

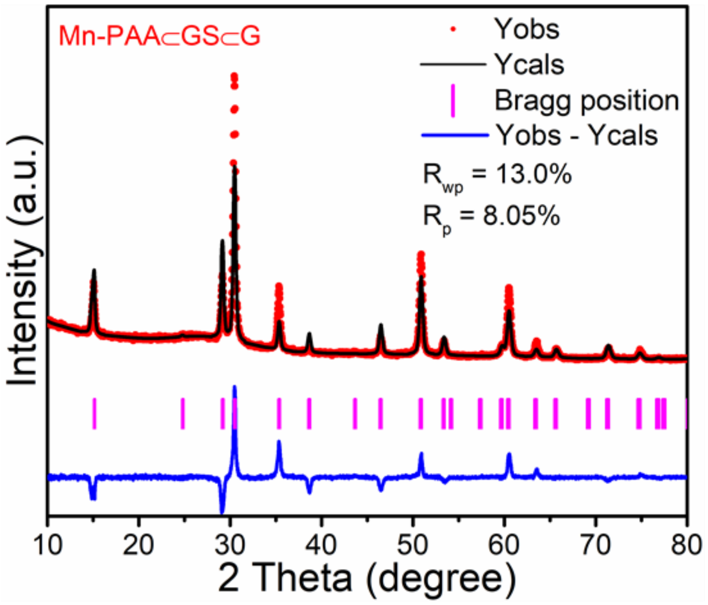

Figure S17 The Rietveld refinement result of the Mn-PAA⊂GS⊂G when the Mn^{2+} ions are fixed on 16c position of the pyrochlore structure.

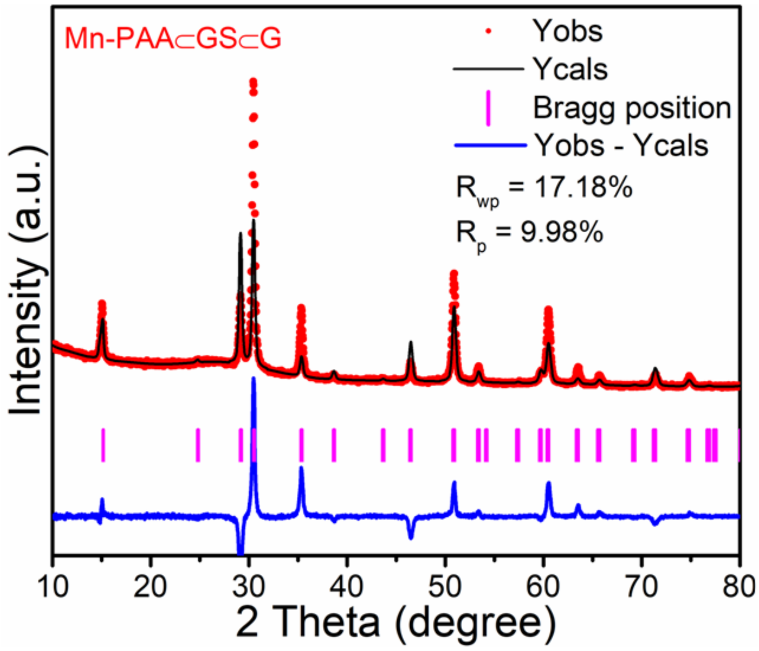

Figure S18 The Rietveld refinement result of the Mn-PAA⊂GS⊂G when the Mn^{2+} ions are fixed on 8b position of the pyrochlore structure.

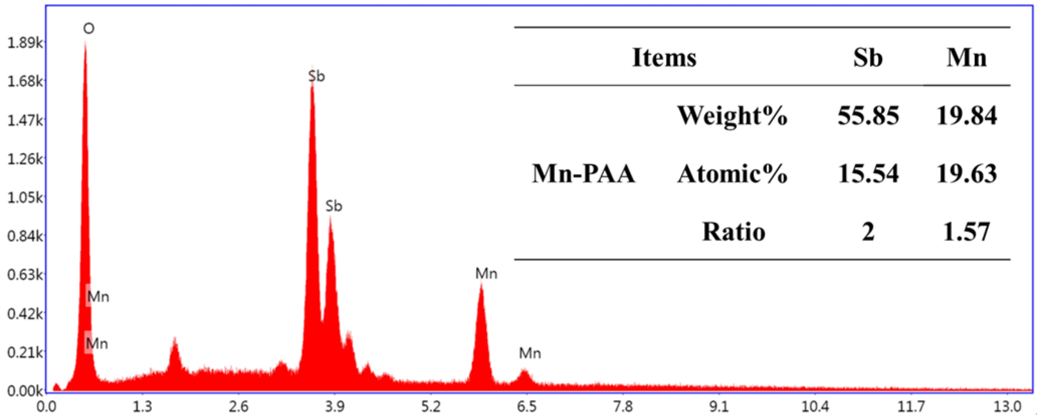

Items		Sb	Mn
	Weight%	55.85	19.84
Mn-PAA	Atomic%	15.54	19.63
	Ratio	2	1.57

Figure S19 EDX results of the Mn-PAA.

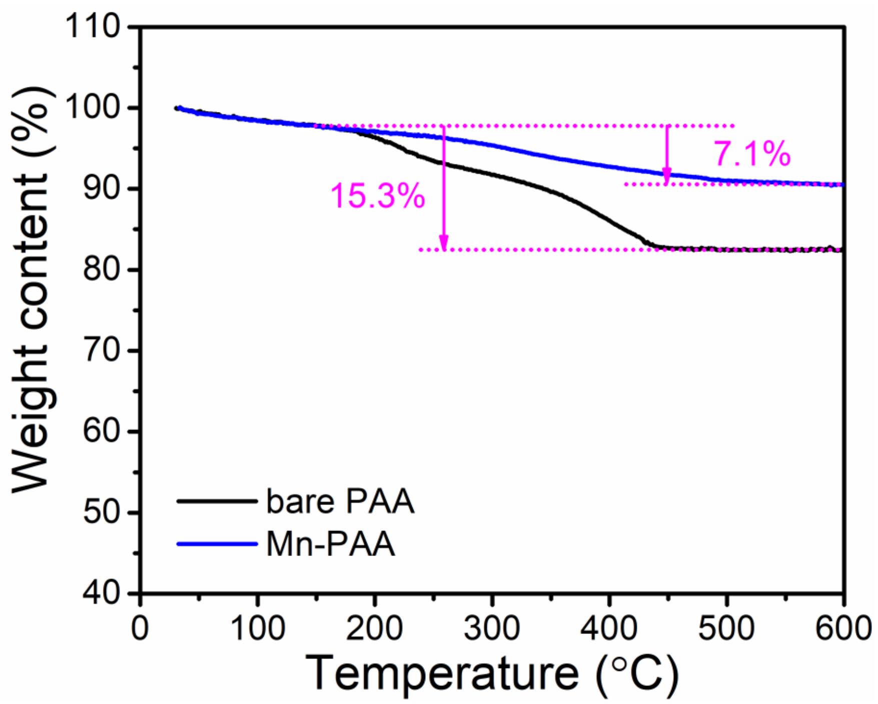

Figure S20 TG curve of the bare PAA and Mn-PAA sample in N_2 atmosphere.

Table S4 ICP-OES results of two Mn-PAA samples.

**Samples**	Mn 257.6 nm (mg L^{-1})	Sb 217.6 nm (mg L^{-1})	Ratio (Mn: Sb)
**#1**	5.70	7.19	1.59: 2
**#2**	5.92	7.40	1.60: 2

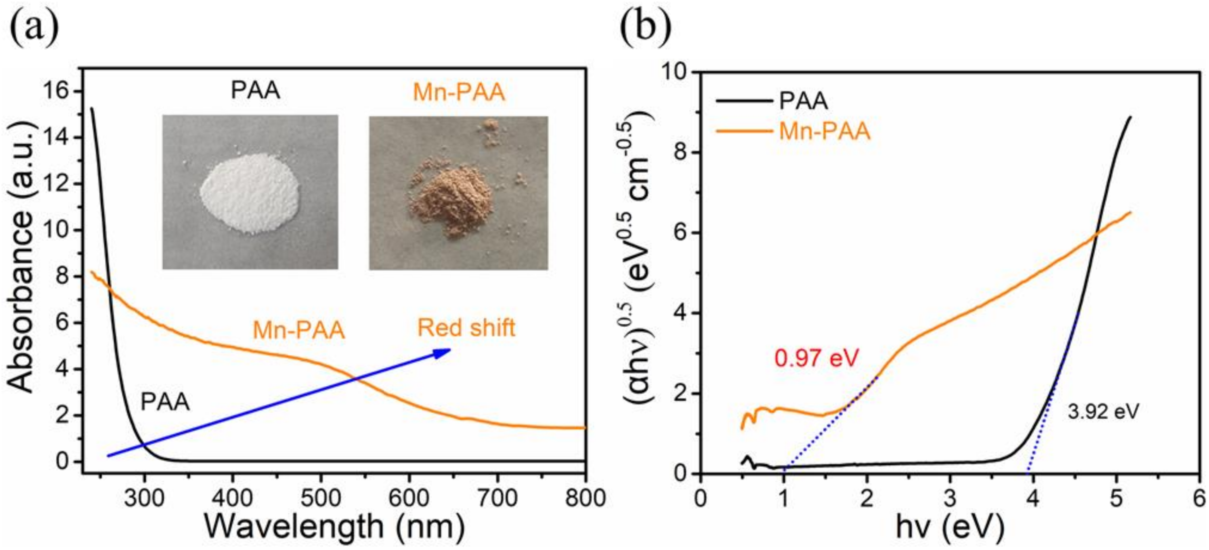

Figure S21 (a) UV-vis DRS spectra and (b) Tauc plots of the PAA and Mn-PAA.

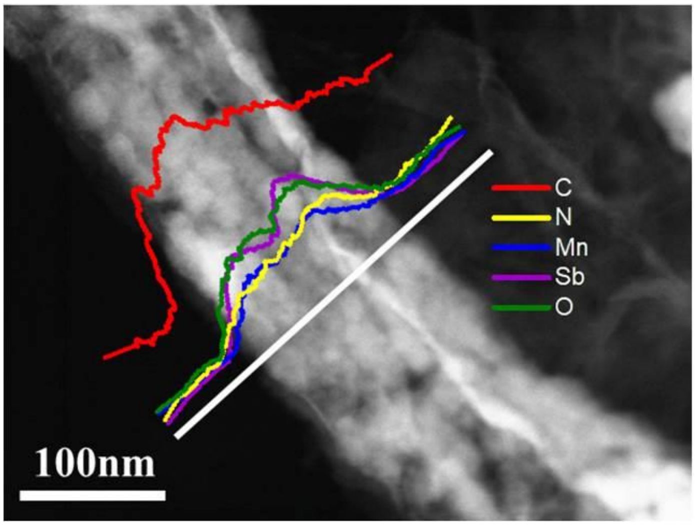

Figure S22 HAADF-STEM image and corresponding cross-sectional EDX line scanning profile of the Mn-PAA⊂GS⊂G.

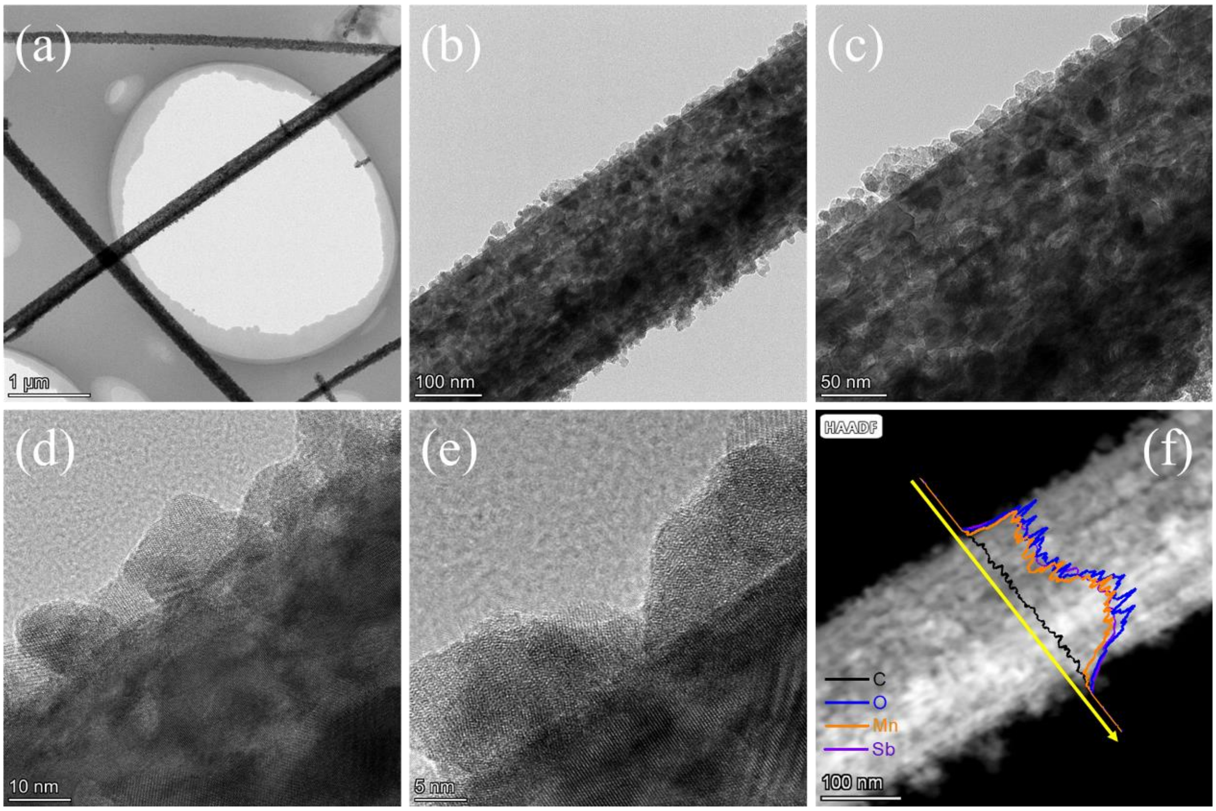

Figure S23 TEM, HRTEM, and EDX line scanning images of the MnO_2@Mn-PAA suffered PDDA treatment and then annealed at 400 °C for 2 h in argon atmosphere.

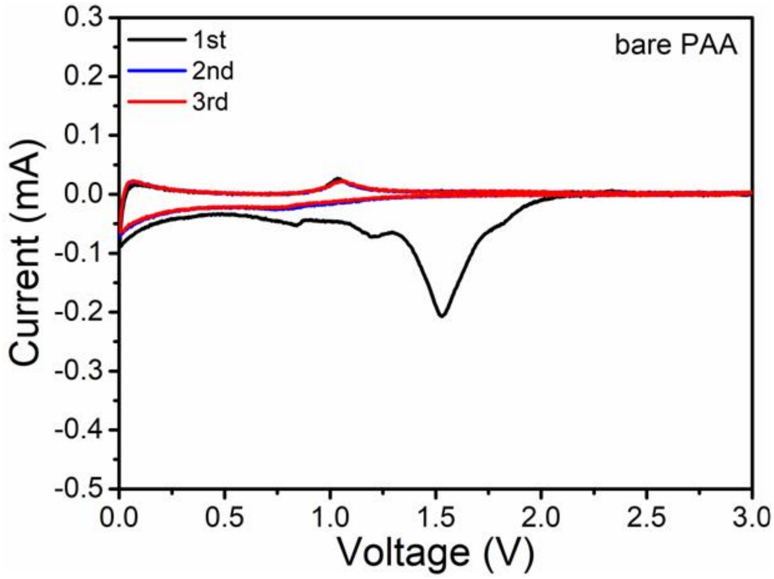

Figure S24 CV curves of the bare PAA electrode.

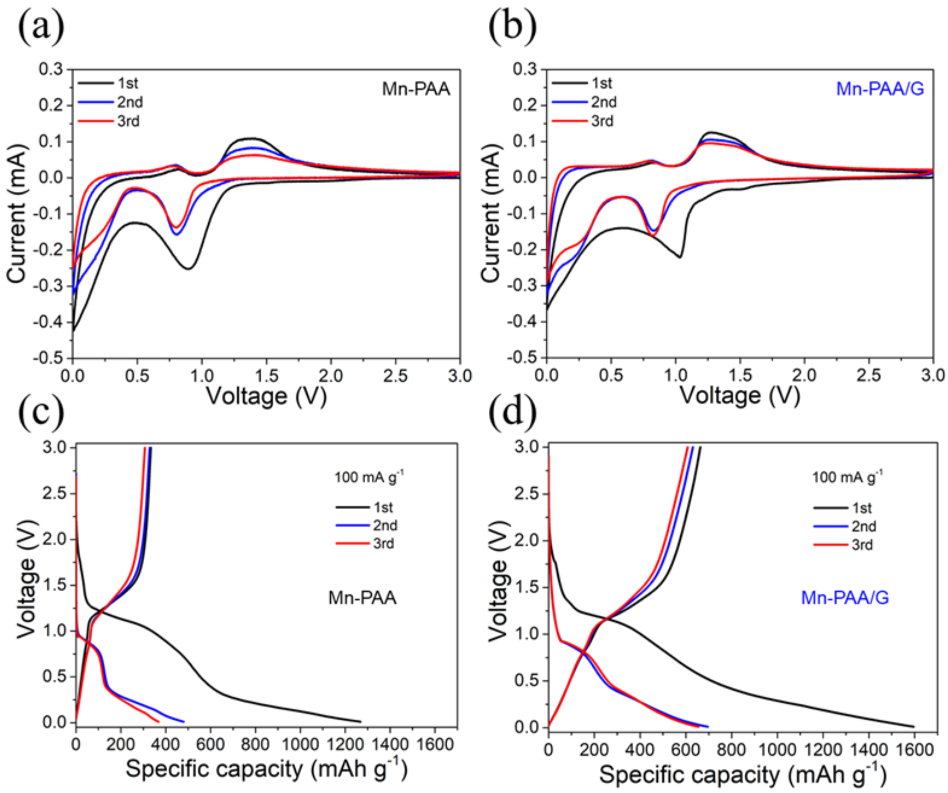

Figure S25 CV curves of the (a) Mn-PAA and (b) Mn-PAA/G; Initial three discharge/charge profiles of the (c) Mn-PAA and (d) Mn-PAA/G.

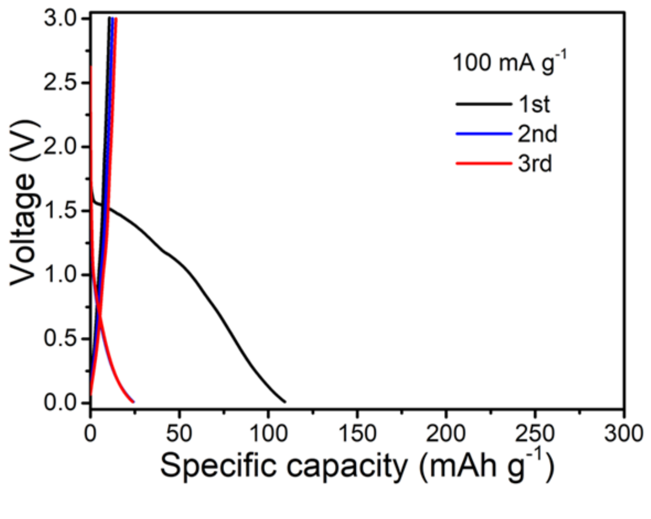

Figure S26 Initial three discharge/charge profiles of the bare PAA.

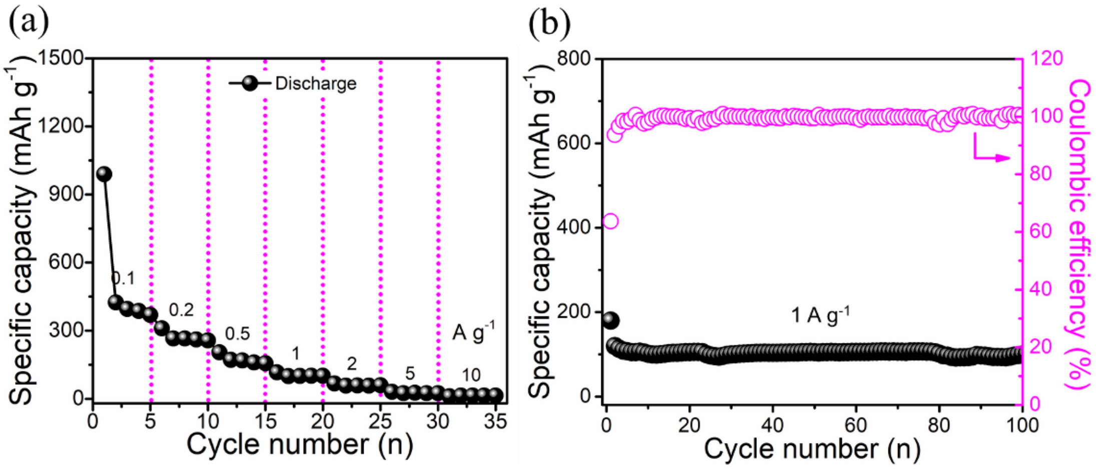

Figure R27 Rate capability and cycling performance of the GS⊂G anode material for LIBs.

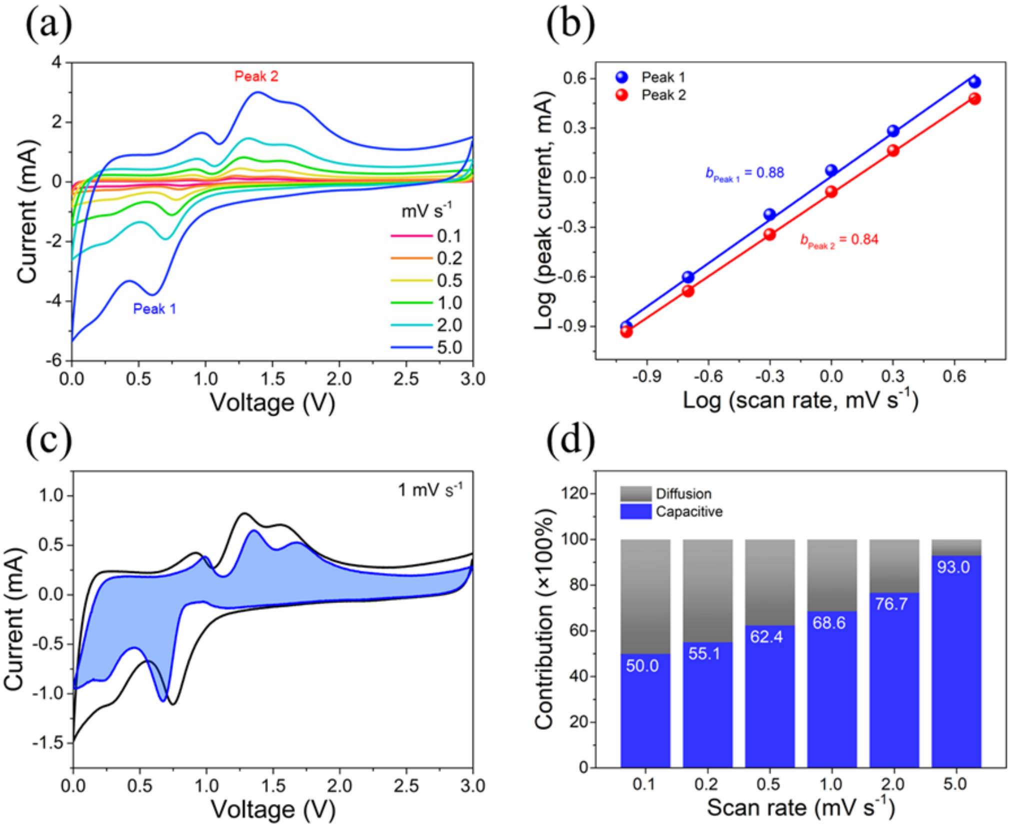

Figure S28 (a) CV curves of the Mn-PAA⊂GS⊂G at different scan rates of 0.1, 0.2, 0.5, 1.0, 2.0, and 5.0 mV s^{-1}; (b) *b* values of the corresponding anodic and cathodic CV processes; (c) The estimated capacitive current contribution of the Mn-PAA⊂GS⊂G at

a scan rate of 1 mV s^{-1}; (d) The capacitive contribution ratio in the total intercalated charge as a function of scan rates.

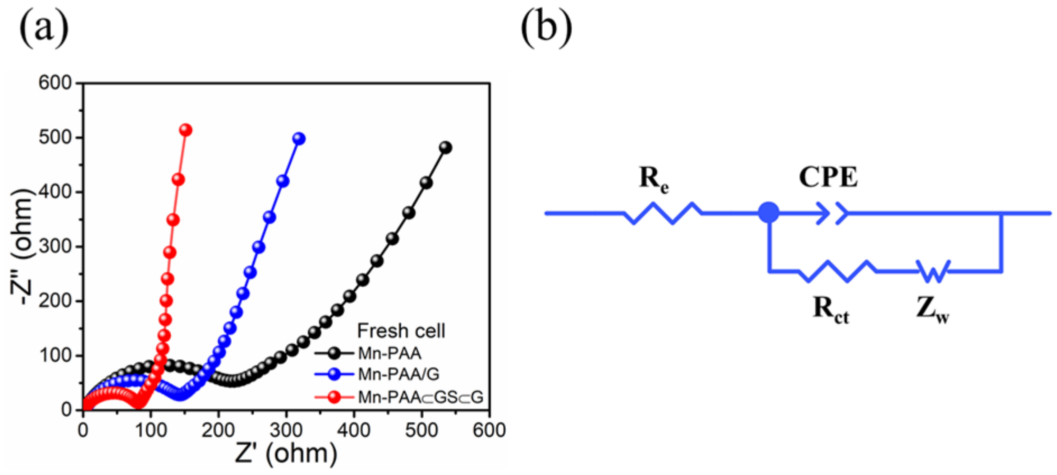

Figure S29 (a) EIS spectra of the Mn-PAA, Mn-PAA/G, and Mn-PAA⊂GS⊂G electrodes; (b) Equivalent circuit model used to fit cells. R_e: electrolyte resistance (including the bulk resistance in the electrolyte, separator, and electrode); CPE: constant phase element reflecting double layer capacitance; R_{ct}: charge transfer resistance; Z_W: Warburg impedance.

Table S5 The fitting parameters of the Mn-PAA, Mn-PAA/G, and Mn-PAA⊂GS⊂G electrodes.

**Samples**	R_e (Ω)	R_{ct} (Ω)	R_{all} (Ω)
Mn-PAA	2.2	235.6	237.8
Mn-PAA/G	2.5	150.5	153.0
Mn-PAA⊂GS⊂G	2.2	81.7	83.9

Table S6 Comparison of the cycling performance of the Mn-PAA⊂GS⊂G with previously reported Sb-O based anode materials for LIBs.

Materials	Current density	Cycle number (n)	Capacity	Retention ratio	Reference
Sb_2O_3/RGO	50 mA g^{-1}	100	562 mAh g^{-1}	63%	[2]
Sb_6O_{13}/RGO	1000 mA g^{-1}	300	290 mAh g^{-1}	47%	[3]
Sb_2O_3@TiO_2	500 mA g^{-1}	600	439 mAh g^{-1}	66%	[4]
Sb_2O_4	1000 mA g^{-1}	100	415 mAh g^{-1}	83%	[5]
Sb_2O_4/RGO	550 mA g^{-1}	500	428 mAh g^{-1}	50%	[6]
Sb_2O_3	200 mA g^{-1}	50	641 mAh g^{-1}	62%	[7]
Sb_2O_3/RGO	600 mA g^{-1}	700	526 mAh g^{-1}	56%	[8]
Sb_2O_3	50 mA g^{-1}	50	277 mAh g^{-1}	80%	[9]
$ZnSb_2O_6$	500 mA g^{-1}	300	400 mAh g^{-1}	100%	[10]
**Mn-PAA⊂GS⊂G**	**1000 mA g^{-1}**	**1000**	**1077 mAh g^{-1}**	**100%**	**This work**

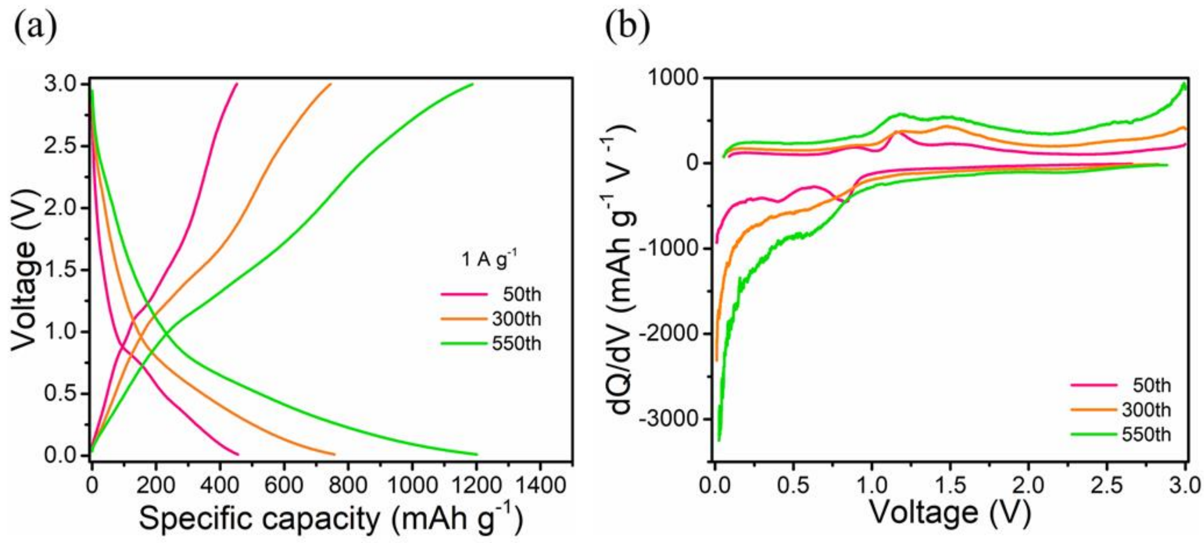

Figure S30 (a) Selected discharge/charge curves and (b) corresponding differential capacity vs. voltage (dQ/dV) plots of the Mn-PAA⊂GS⊂G.

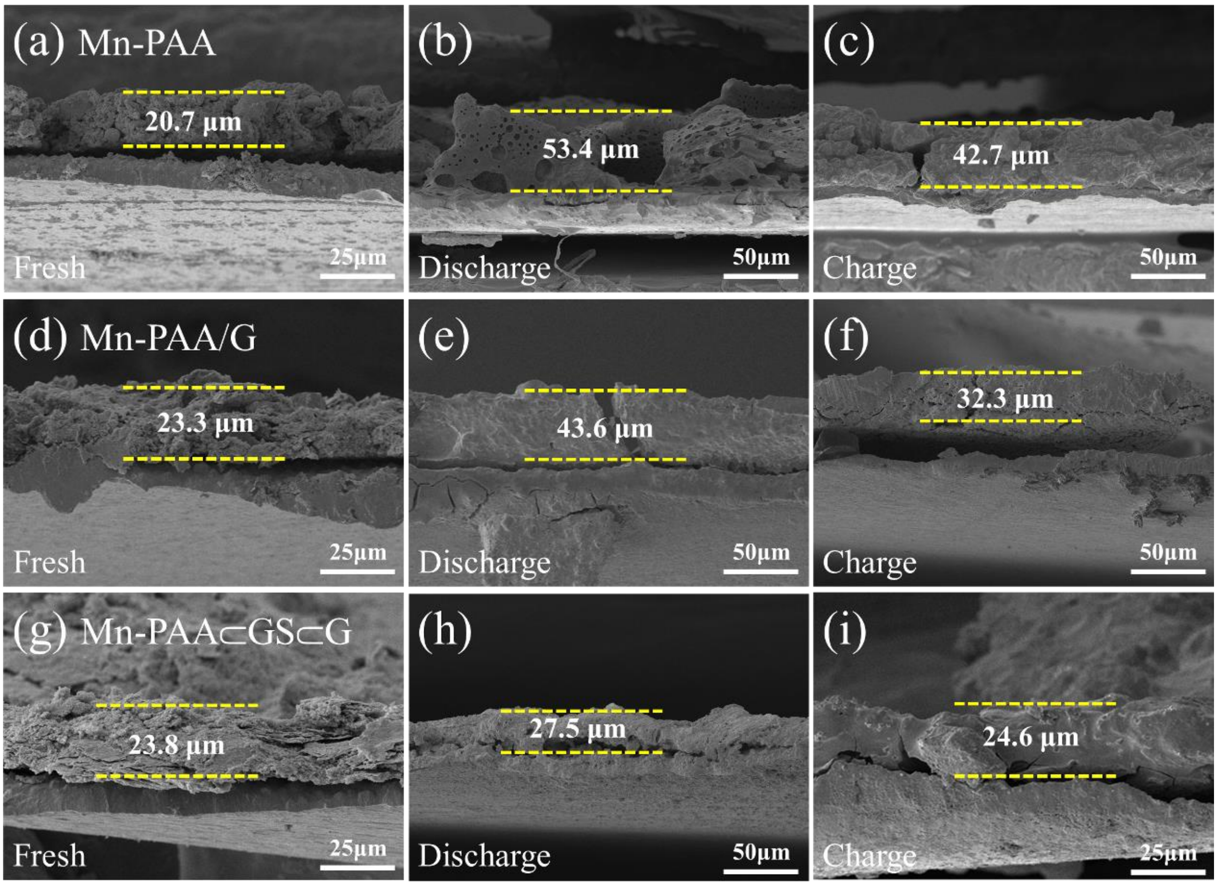

Figure S31 The thickness changes of the Mn-PAA, Mn-PAA/G, and Mn-PAA⊂GS⊂G electrodes at different state of charge.

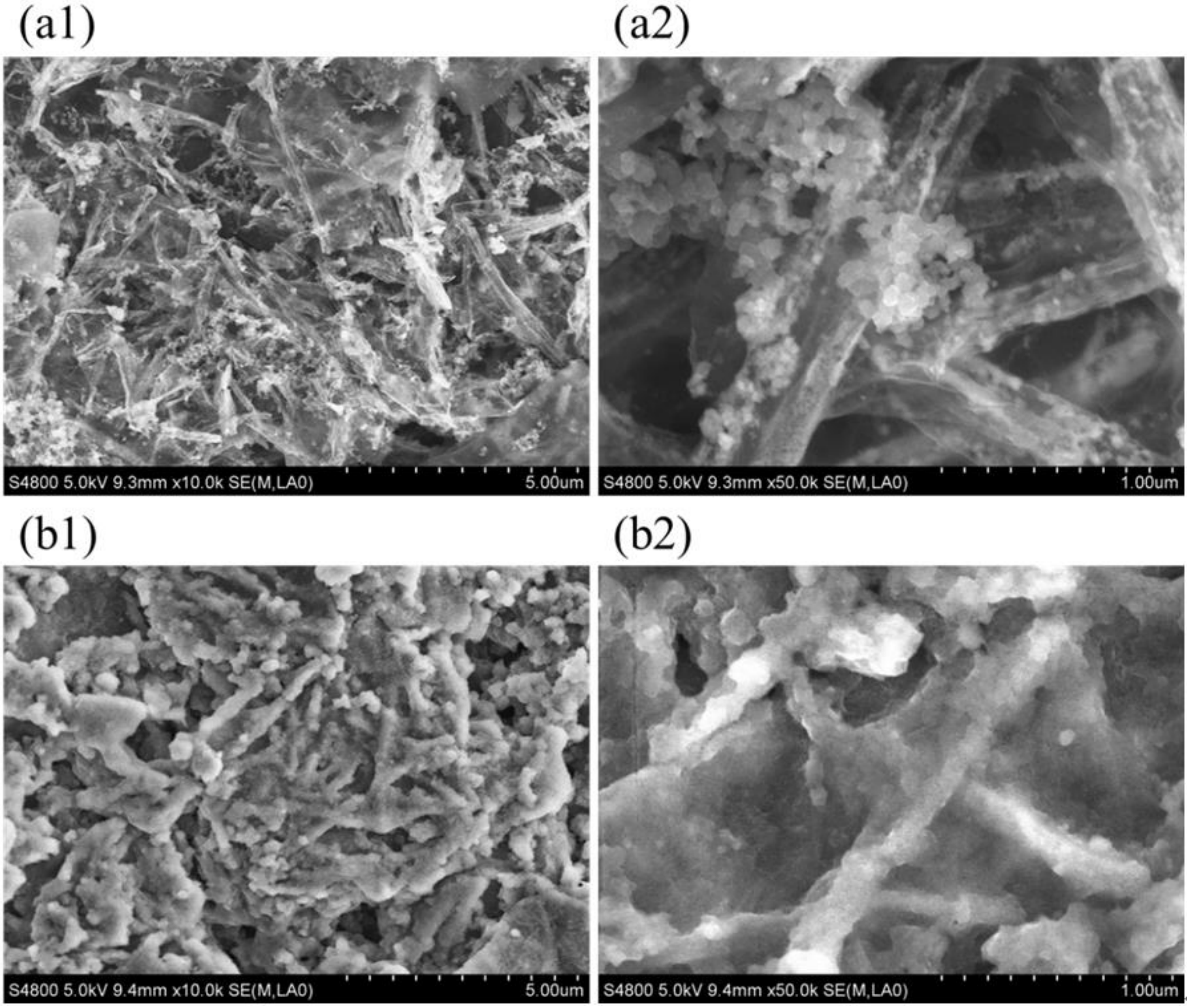

Figure S32 FESEM images of the Mn-PAA⊂GS⊂G electrode before (a1, a2) and after (b1, b2) 100 cycles at 1 A g^{-1}.

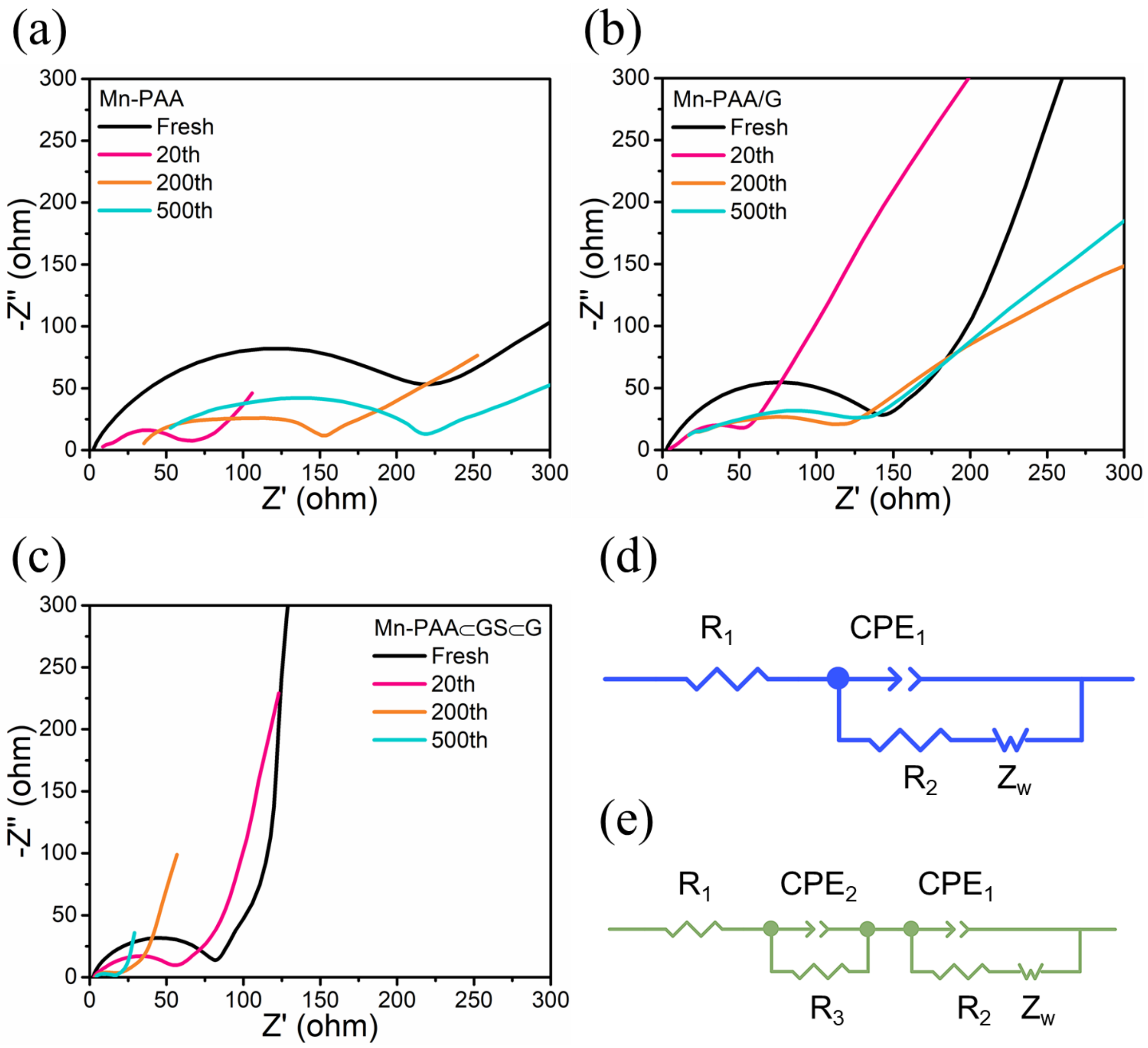

Figure S33 EIS spectra of the Mn-PAA, Mn-PAA/G, and Mn-PAA⊂GS⊂G electrodes at different cycles; (b) Equivalent circuit models used to fit cells. R_1: electrolyte contact resistance, including the bulk resistance in the electrolyte, separator, and electrode; R_2: charge transfer resistance in the interface of materials/electrolyte; R_3: charge transfer resistance in the interface of in the SEI film; CPE: space charge capacitance; Z_W: Warburg impedance.

Table S7 The fitting parameters of the Mn-PAA, Mn-PAA/G, and Mn-PAA⊂GS⊂G electrodes at different cycles.

**Samples**	**Cycles**	$\boldsymbol{R_1}$ **(Ω)**	$\boldsymbol{R_2}$ **(Ω)**	$\boldsymbol{R_3}$ **(Ω)**	$\boldsymbol{R_{all}}$ **(Ω)**
Mn-PAA	Fresh	2.2	235.6	/	**237.8**
	20th	3.1	20.7	36.3	**60.1**
	200th	32.1	29.0	77.5	**138.6**
	500th	39.7	85.1	74.0	**198.8**
Mn-PAA/G	Fresh	2.5	150.5	/	**153.0**
	20th	4.9	50.4	/	**55.3**
	200th	5.9	86.5	/	**92.4**
	500th	6.1	110.3	/	**116.4**
Mn-PAA⊂GS⊂G	Fresh	2.2	81.7	/	**83.9**
	20th	2.5	46.1	/	**48.6**
	200th	3.4	11.6	/	**15.0**
	500th	3.6	11.3	/	**14.9**

References

[1] Y. Zhang, P. Chen, X. Gao, B. Wang, H. Liu, H. Wu, H. Liu, S. Dou, *Adv. Funct. Mater.*, **2016**, 26, 7754.

[2] J. Zhou, C. Zheng, H. Wang, J. Yang, P. Hu, L. Guo, *Nanoscale*, **2016**, 8, 17131.

[3] X. Zhou, Z. Zhang, X. Xu, J. Yan, G. Ma, Z. Lei, *ACS Appl. Mater. Interfaces*, **2016**, 8, 35398.

[4] Z. Wang, Y. Cheng, Q. Li, L. Chang, L. Wang, *J. Power Sources*, **2018**, 389, 214.

[5] Z. Yi, Q. Han, X. Li, Y. Wu, Y. Cheng, L. Wang, *Chem. Eng. J*, **2017**, 315, 101.

[6] X. Zhou, Z. Zhang, J. Wang, Q. Wang, G. Ma, Z. Lei, *J. Alloy. Compd.*, **2017**, 699, 611.

[7] M. Deng, S. Li, W. Hong, Y. Jiang, W. Xu, H. Shuai, G. Zou, Y. Hua, H. Hou, W. Wang, X. Ji, *Mater. Chem. Phys.*, **2019**, 223, 46.

[8] X. Zhou, Z. Zhang, X. Lu, X. Lv, G. Ma, Q. Wang, Z. Lei, *ACS Appl. Mater. Interfaces*, **2017**, 9, 34927.

[9] Y. Tan, L. Chen, H. Chen, Q. Hou, X. Chen, *Mater. Lett.*, **2018**, 212, 103.

[10] J. Li, K. Du, Y. Lai, Y. Chen, Z. Zhang, *J. Mater. Chem. A*, **2017**, 5, 10843.
